# Ca^2+^ enrichment in culture medium potentiates effect of oligonucleotides

**DOI:** 10.1093/nar/gkv626

**Published:** 2015-06-22

**Authors:** Shin-ichiro Hori, Tsuyoshi Yamamoto, Reiko Waki, Shunsuke Wada, Fumito Wada, Mio Noda, Satoshi Obika

**Affiliations:** Graduate School of Pharmaceutical Sciences, Osaka University, 1–6, Yamadaoka, Suita, Osaka, 565-0871, Japan

## Abstract

Antisense and RNAi-related oligonucleotides have gained attention as laboratory tools and therapeutic agents based on their ability to manipulate biological events *in vitro* and *in vivo*. We show that Ca^2+^ enrichment of medium (CEM) potentiates the *in vitro* activity of multiple types of oligonucleotides, independent of their net charge and modifications, in various cells. In addition, CEM reflects *in vivo* silencing activity more consistently than conventional transfection methods. Microscopic analysis reveals that CEM provides a subcellular localization pattern of oligonucleotides resembling that obtained by unassisted transfection, but with quantitative improvement. Highly monodispersed nanoparticles ∼100 nm in size are found in Ca^2+^-enriched serum-containing medium regardless of the presence or absence of oligonucleotides. Transmission electron microscopy analysis reveals that the 100-nm particles are in fact an ensemble of much smaller nanoparticles (ϕ ∼ 15 nm). The presence of these nanoparticles is critical for the efficient uptake of various oligonucleotides. In contrast, CEM is ineffective for plasmids, which are readily transfected via the conventional calcium phosphate method. Collectively, CEM enables a more accurate prediction of the systemic activity of therapeutic oligonucleotides, while enhancing the broad usability of oligonucleotides in the laboratory.

## INTRODUCTION

The development of robust technologies to decipher the roles of yet-to-be-annotated cellular transcripts and proteins is a major challenge. Ideally, such technologies should be achieved concomitantly with therapeutic manipulation of these factors. Oligonucleotide-based technologies (such as antisense oligonucleotides (ASOs), aptamers, siRNAs, anti-miRNAs and more recently single guide RNAs (sgRNAs) for CRISPR-Cas9 systems) have increasingly gained attention in terms of their extraordinary ability to precisely recognize biological targets in a sequence-dependent and/or -independent manner. These methodologies have permitted successful modification of gene expression and protein activity *in vitro* to elucidate biological and pathological mechanisms. In addition, recent innovations in the oligonucleotide chemistry have also improved the potency and pharmacokinetics of oligonucleotide-based therapeutics (1–3). These modifications provide robust systemic oligonucleotide activity even in the absence of delivery vehicles, directly expanding their applicability as therapeutic agents. However, we still lack appropriate *in vitro* systems that can predict *in vivo* oligonucleotide activity and toxicity during drug discovery and development. In fact, the *in vitro* evaluation of therapeutic oligonucleotides is typically still conducted using conventional carrier-dependent transfection; such methods often yield false-positives (4,5). In addition, conventional techniques often involve multiple cumbersome steps (e.g., invisible complex formation), cytotoxicity of delivery materials and an enormous repertoire of vehicles, among which appropriate vehicles have to be chosen in accordance with specific oligonucleotide chemistries. Several alternative *in vitro* systems have been developed to date. Cultured primary cells exhibit efficient uptake of naked oligonucleotides associated with potent gene silencing, however, this uptake is lost 24–36 h after cell isolation (5,6). Koller *et al*. successfully maintained the ability of primary cells to efficiently take up naked chemically-modified oligonucleotides by establishing a mouse hepatocellular SV40 large T-antigen carcinoma cell line (MHT) from a specific transgenic mouse (7); however, this transgenic technique is not appropriate for human applications. Most notably, systemically active naked oligonucleotides modified with 2′,4′-bridged nucleic acid (2′,4′-BNA) (also known as locked nucleic acid, LNA) chemistry (8,9) have recently been found to be taken up by various cell lines, including human cells, without the use of transfection reagents (10). Interestingly, the *in vitro* free-uptake of these biologically stable oligonucleotides was shown to reflect *in vivo* activity (4,10,11). However, despite this clear advantage, the efficiency of free-uptake varies widely depending on the host cell line and also requires long-term exposure to medium, limiting the application of free-uptake to biologically stable oligonucleotides.

Ca^2+^ ions have been widely used in gene transfection, a role facilitated by the formation of co-precipitates between plasmid DNA and calcium phosphate (12). However, this method is rarely used for oligonucleotides and requires a delicate multistep process for the manipulation of fragile precipitates, in sharp contrast to the simple method described below (13). On the other hand, Ca^2+^ has been reported to enhance the transfection efficiency of polycations (such as histone H1 protein, high-mobility group protein-1 (HMG1) complexed with plasmid DNAs and cationic peptide-conjugated peptide nucleic acids (PNAs) (14–16)), although the mechanism of the Ca^2+^ effect on transfection remains largely unknown. Based on these previous observations, we speculate that Ca^2+^ enrichment in medium, hereafter referred to as the ‘CEM method’, has a role in delivering oligonucleotides inside cells, independent of the oligonucleotides’ charge and chemical modifications.

We report here that an alkaline-earth metal salt, calcium chloride (CaCl_2_), potentiates the activity of multiple types of naked oligonucleotides, but not that of plasmids, in various cultured cell lines with limited cytotoxicity. The *in vitro* potency of oligonucleotides obtained with simple culture medium enrichment with Ca^2+^ is shown to have a higher positive correlation with *in vivo* activity compare to a conventional technique. We anticipate our method will enable more rapid and accurate *in vitro* screening for therapeutic oligonucleotides and may prove to be a powerful laboratory technique for modulating gene expression.

## MATERIALS AND METHODS

### Oligonucleotides

All 2′,4′-BNA/LNA-based ASOs were synthesized and purified by Gene Design, Inc. (Osaka, Japan). *ApoB* siRNAs, ZsGreen1 siRNA and negative control siRNA, with a medium GC content, were obtained from Invitrogen (Carlsbad, CA, USA) as Stealth RNAi siRNAs. ST6-PMO and NC-PMO were purchased from Gene-Tools, LLC (Corvallis, OR, USA). The sequences of ASOs, siRNAs and PMOs used in this study are shown in Supplementary Tables S1–S3.

### Cell culture

Huh-7, HLE, HeLa, HEK293 and A549 cells were obtained from the Japanese Collection of Research Bioresources (JCRB; Osaka, Japan). Plat-GP packaging cells were purchased from Cosmo Bio (Tokyo, Japan). All cell lines were maintained at 37°C and 5% CO_2_ in Dulbecco's Modified Eagle's Medium (DMEM; Nacalai Tesque, Kyoto, Japan) supplemented with 10% heat-inactivated fetal bovine serum (FBS) and antibiotics.

### Plasmid construction and preparation of stable cell lines expressing ZsGreen1 and DsRed

To generate a retroviral vector expressing ZsGreen1, pZsGreen1-N1 (TaKaRa Bio Inc., Shiga, Japan) was digested with Bam HI and Not I and ligated into the Bam HI- and Not I-digested pENTR2B (Invitrogen). Subsequently, the ZsGreen1 fragment was transferred to the retroviral expression vector pQCXIN (TaKaRa Bio Inc.), which was converted into a destination vector using LR clonase enzyme mix (Invitrogen). The production of recombinant retroviruses expressing ZsGreen1 or DsRed was performed according to standard protocols. Briefly, the Plat-GP packaging cells were cotransfected with the recombinant retroviral vector pQCXIN expressing ZsGreen1 or pRetroQ-DsRed-N1 (TaKaRa Bio Inc.) and pCMV-VSV-G (Cosmo Bio) by using Fugene6 (Promega, Madison, WI, USA). Viral supernatants were harvested 48 h after transfection, filtered through 0.45-μm filters and subjected to ultracentrifugation at 50 000 × *g* at 4°C for 2 h prior to use as viral stocks. HLE cells were infected with the viral stock expressing ZsGreen1 in DMEM with 10 μg/ml Polybrene^®^ (hexadimethrine bromide) and the infected cells were selected in the presence of 400 μg/ml G418. Subsequently, HLE cells expressing ZsGreen1 were infected with the viral stock expressing DsRed and the doubly-infected cells, which we designated ZsG-N1–2R/HLE cells, were selected in the presence of 400 μg/ml G418 and 1 μg/ml puromycin.

### Evaluation of knockdown activity of ZsGreen1-ASO in ZsG-N1–2R/HLE cells

**Figure 1.**
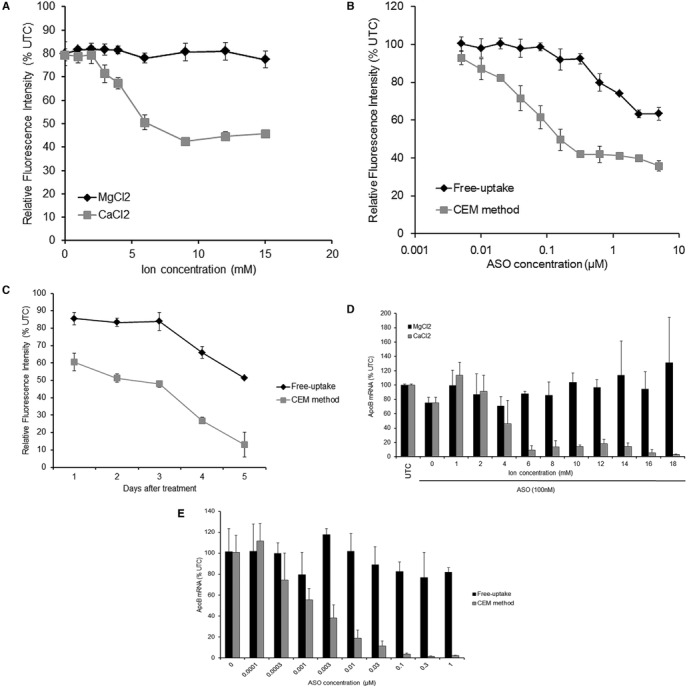
CEM effect on the activity of 2′, 4′-BNA/LNA-modified ASOs. (**A**) Effect of ion concentration on the activity of ZsGreen1-ASO in ZsG-N1–2R/HLE cells. ZsG-N1–2R/HLE cells were treated with ZsGN1–120-BNA (15) at 300 nM for 4 days in the presence of MgCl_2_ or CaCl_2_. Relative fluorescence intensity (RFI) was measured and is presented as the percentage relative to the untreated control (UTC). (**B**) ZsG-N1–2R/HLE cells were treated with ZsGN1–120-BNA (15) at concentrations ranging from 5 nM to 5 μM for 4 days in the presence or absence of 9 mM CaCl_2_. RFI was measured and is presented as the percentage relative to the UTC. (**C**) Time course of change in the knockdown activity of ZsGreen1-ASO. ZsG-N1–2R/HLE cells were treated with ZsGN1–120-BNA (15) at 1 μM for 5 days in the presence or absence of CaCl_2_. RFI was measured every 24 h for 5 days. (**D**) Effect of ion concentration on the activity of *ApoB*-ASO in Huh-7 cells. Huh-7 cells were treated with *ApoB*-ASO at 100 nM for 24 h in the presence of MgCl_2_ or CaCl_2_. Total RNA was extracted and *ApoB* mRNA was quantitated by qRT-PCR. The relative quantification of *ApoB* mRNA was normalized against the expression of the *GAPDH* gene. (**E**) Dose dependency of *ApoB*-ASO. Huh-7 cells were treated with *ApoB*-ASO at concentrations ranging from 0.1 nM to 1 μM for 24 h in the presence or absence of 9 mM CaCl_2_. Total RNA was extracted and *ApoB* mRNA was quantitated by qRT-PCR. The relative quantification of *ApoB* mRNA was normalized against the expression of the *GAPDH* gene. Each data point represents the mean ± SD of three independent experiments.

ZsG-N1–2R/HLE cells were seeded at 1.2 × 10^4^ cells/well in 96-well black plates (Corning; Corning, NY, USA) containing 10% FBS/DMEM. After 24 h, each ZsGreen1-ASO was added in 10% FBS/DMEM containing or lacking CaCl_2_ or MgCl_2_. In separate experiments, ZsGN1–120-BNA (15) was used either at 300 nM for 4 days (Figure 1A), from 5 nM to 5 μM for 4 days (Figure 1B) or at 1 μM for 5 days (Figure 1C). To examine if CEM affects the uptake and/or intracellular trafficking of ASO, CaCl_2_ was added to the medium before addition of ZsGN1–120-BNA (15) (‘Pre’ interval) and/or together with addition of ASO for 7 h (‘TF’ interval; expected to be the time of ASO uptake), and/or after removal of ASO and washing of the cells (‘Post’ interval). At 72 h after ASO addition, the knockdown activity of ASO was analyzed (Figure 4B). The fluorescence of ZsGreen1 and DsRed was measured using a microplate spectrofluorometer (SPECTRAmax GEMINI; Molecular Devices, Sunnyvale, CA, USA). Knockdown efficiency of ASO was calculated by dividing the fluorescence of ZsGreen1 by that of DsRed; relative fluorescence intensity (RFI) is presented as the percentage relative to the untreated control (UTC).

To compare the knockdown activity of each ASO between transfection methods, ZsG-N1–2R/HLE cells were treated with one of fourteen different ZsGreen1-ASOs (Supplementary Table S1) at 2.5 μM for 4 days in the presence of CaCl_2_ or at 5 μM for 6 days in the absence of CaCl_2_. Lipofection was performed using RNAiMAX (Invitrogen) according to manufacturer's protocol. After 48 h of lipofection with 20 nM ZsGreen1-ASOs, the fluorescence of ZsGreen1 and DsRed was measured and knockdown activity was calculated as above.

### Evaluation of *in vitro* activity of ASO or siRNA

**Figure 2.**
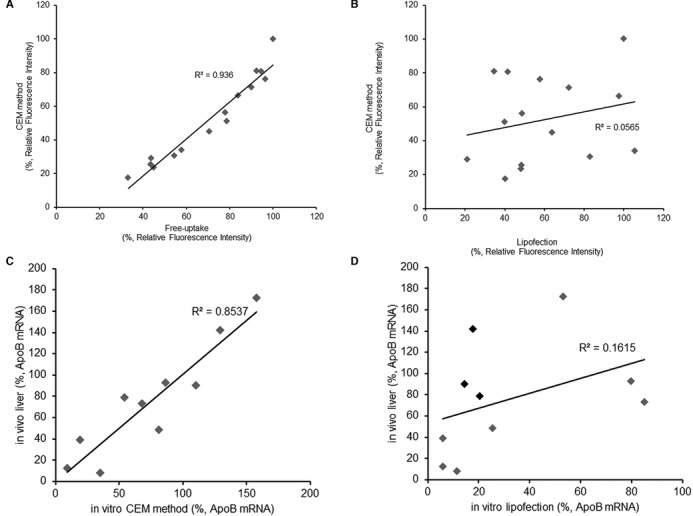
CEM method reflects the activity of antisense oligonucleotides delivered by free-uptake and *in vivo* silencing activity more strictly than does lipofection. (**A**) ZsG-N1–2R/HLE cells were treated with each of 14 different ZsGreen1-ASOs at 2.5 μM in the presence of CaCl_2_ in the medium. After 4 days, the fluorescence of ZsGreen1 and DsRed were measured. To compare the knockdown activity of each ASO with free-uptake, ZsG-N1–2R/HLE cells were treated with each respective ZsGreen1-ASO at 5 μM in the absence of CaCl_2_ in the medium. After 6 days, the fluorescence of ZsGreen1 and DsRed were measured. Each data point represents the mean of three independent experiments. The mean value of knockdown activity was plotted in the two-dimensional space to analyze the correlation between transfection methods. (**B**) To compare the knockdown activities to those obtained by lipofection, ZsG-N1–2R/HLE cells were lipofected using RNAiMAX (Invitrogen). After 48 h of transfection, the fluorescence of ZsGreen1 and DsRed were measured and knockdown activity was calculated. Each data point represents the mean of three independent experiments. The mean value of knockdown activity was plotted in the two-dimensional space to analyze the correlation between transfection methods. (**C** and **D**) To compare the *in vitro* activity of CEM and lipofection with *in vivo* activity, Huh-7 cells were treated with each of ten different *ApoB*-ASOs at 10 nM in the presence of 9 mM CaCl_2_ in the medium or transfected using RNAiMAX. After 24 h, total RNA was extracted and *ApoB* mRNA was quantitated by qRT-PCR. Mice (C57BL/6, *n* = 3/group) were injected subcutaneously (at 10 mg/kg) with a single dose of each of 10 *ApoB*-ASOs. Mice were sacrificed 72 h later and livers were analyzed for reductions in *ApoB* mRNA levels. The relative quantification of *ApoB* mRNA was normalized against the expression of the *GAPDH* gene. Each data point represents the mean of three independent experiments. The mean value of knockdown activity was plotted in the two-dimensional space to analyze the correlation between transfection methods.

Huh-7 cells were seeded at 8.0 × 10^3^ cells/well in 96-well plates (Corning) containing 10% FBS/DMEM. After 24 h, ApoB-10177-BNA (13) at either 100 nM (Figure 1D) or from 0.1 nM to 1 μM (Figure 1E) or one of the *ApoB*-siRNAs at 1 μM (Figure 5A) was added, and cells were cultured in medium with or without CaCl_2_. To compare the activity of 10 different *ApoB*-ASOs, the ASOs were used at 10 nM in the presence of 9 mM CaCl_2_ (Figure 2C) or transfected with RNAiMAX (Invitrogen) according to the manufacturer's protocol. After 24 h, total RNA was extracted with a CellAmp Direct RNA Prep Kit (Takara Bio Inc.) according to the manufacturer's instructions. Quantitative reverse transcription-polymerase chain reaction (qRT-PCR) was performed with a One Step SYBR PrimeScript PLUS RT-PCR Kit (Takara Bio Inc.) and analyzed with a StepOnePlus system (Applied Biosystems; Foster City, CA, USA). The primers used in this study were specific for the human *ApoB* gene (forward: 5′-TTCTCAAGAGTTACAGCAGATCCA-3′; reverse: 5′-TGGAAGTCCTTAAGAGCAACTAACA-3′) and for the human *GAPDH* gene (forward: 5′-GCACCGTCAAGGCTGAGAAC-3′; reverse:5′-TGGTGAAGACGCCAGTGGA-3′). The level of target (*ApoB*) gene expression was normalized to that of *GAPDH*.

### *In* *vivo* experiment

All animal experimentation protocols were approved by the Institutional Animal Care and Use Committee of Osaka University. Male C57BL/6J mice, 7-weeks-old, were obtained from CLEA Japan (Tokyo, Japan). Mice were maintained on a 12-h light/dark cycle and allowed food and water *ad libitum*. Each mouse received a single subcutaneous injection at 10 mg/kg of one of the *ApoB*-ASOs. At 72 h after administration, mice were anesthetized with isoflurane (Abbott Laboratories, North Chicago, IL, USA) and sacrificed. Livers were then harvested, snap frozen in liquid nitrogen, and stored at −80°C until use. Total RNA was isolated from frozen liver tissue using QuickGene RNA tissue kit SII (FujiFilm, Tokyo, Japan) according to the manufacturer's instructions. qRT-PCR was performed with a High Capacity cDNA Reverse Transcription kit (Life Technologies, Gaithersburg, MD, USA) and analyzed with a StepOnePlus system (Applied Biosystems). The *ApoB* mRNA level was normalized to *GAPDH*. The primers used in this study were specific for the murine *ApoB* gene (forward: 5′- TCCTCGGTGAGTTCAATGACTTTC -3′; reverse: 5′- TGGACCTGCTGTAGCTTGTAGGA -3′) and the murine *GAPDH* gene (forward: 5′- TGTGTCCGTCGTGGATCTGA -3′; reverse: 5′- TTGCTGTTGAAGTCGCAGGAG -3′).

### Confocal microscopy

Huh-7 cells were seeded at 4.5 × 10^4^ cells/well in 8-well covered glass dishes (SCC-038; Mastunami Glass, Osaka, Japan) containing 10% FBS/DMEM without phenol red. After 24 h incubation in a 5% CO_2_ incubator, 500 nM Cy3-labeled ApoB-10177-BNA (13) was added in the presence or absence of 9 mM CaCl_2_ and cultures were incubated for 24 h. For comparison of transfection methods, Cy3-labeled ApoB-10177-BNA (13) was transfected at 100 nM into Huh-7 cells with Lipofectamine 2000 (Life Technologies) or CalPhos Mammalian Transfection Kit (Z1312N; Takara Bio Inc.) according to the manufacturer's procedure. After 24-h incubation, the subcellular localization of Cy3-labeled *ApoB*-ASO was visualized by confocal microscopy. Nuclei were then stained with 0.5 μM Hoechst 33342 (Life Technologies) for 30 min, followed by staining of lysosomes with 75 nM LysoTraker^®^ Green DND-26 (Life Technologies) for 30 min at 37°C. After staining, the medium was replaced with HBSS and the cells were observed using a confocal laser scanning microscope (TCS SP5; Leica). Detection conditions were as follows: objective lens: 40×/Oil, HCX PL APO; Cy3 detection: Ex 543-nm laser (50% power), PMT range 555–700 nm; LysoTracker: Ex 488-nm laser (40% power), PMT range 500–535 nm; Hoechst 33342: Ex 405-nm laser (70% power), PMT range 413–429 nm; DIC: Ex 488-nm (7% power). All images were obtained with a 100 Hz scan speed and averaged across six images. The images were processed with ImageJ software (National Institutes of Health, Bethesda, MD, USA).

### Flow cytometric analysis of ASO uptake

Huh-7 cells were plated at 2 × 10^5^ cells/well in 24-well plates containing 10% FBS/DMEM without phenol red. After 24 h, Cy3-conjugated ApoB-10177-BNA (13) was added to 200 nM in the presence or absence of 9 mM CaCl_2_. After 2-h incubation at 37°C, the cells were washed twice with phosphate-buffered saline (PBS) and detached with trypsin-ethylenediaminetetraacetic acid solution for 5 min at 37°C. Trypsinization was stopped by adding 10% FBS/DMEM without phenol red. Cells were converted into a single-cell suspension by passage through a cell strainer (BD Biosciences, San Jose, CA, USA), and fluorescence intensity of Cy3 in Huh-7 cells was determined using a FACSCalibur with CellQuest Pro software (BD Biosciences).

### Analysis of the calcium effect on PMO activity

Huh-7 cells were seeded in 10% FBS/DMEM in 96-well plates (for RT-PCR) or 12-well plates (for western blotting). After 24-h incubation, individual phosphorodiamidate morpholino oligonucleotides (PMOs) were added at 20 μM in the presence or absence of 9 mM CaCl_2_ in the medium. Endo-Porter (Gene-Tools, LLC, Philomath, OR, USA) was used according to the manufacturer's instructions as a positive control of PMO delivery. After 48 h, RNA extraction and qRT-PCR analysis was performed as above. The sequences of primers used in this study were as follows: hSTAT3-E24-F, 5′- GGAATCCCGTGGGTTGCTTAC-3′ and hSTAT3-E24-R, 5′- TTGAATGCAGTGGCCAGGAC-3′. To confirm the skipping of exon 6, PCR was performed using the following primer set: hSTAT3-E5-F, 5′- TGGTGACGGAGAAGCAGCAGAT-3′ and hSTAT3-E7-R, 5′- TGCACGTACTCCATCGCTGACAAA -3′. The PCR products were analyzed on a 10% Tris-borate-EDTA (TBE) gel stained with ethidium bromide and visualized using an ImageQuant LAS 4000 (Fuji Film). For analysis of protein expression, cells were lysed in Radio-Immunoprecipitation Assay (RIPA) buffer (Sigma-Aldrich, St Louis, MO, USA) with Complete Protease Inhibitor Cocktail (Roche, Indianapolis, IN, USA). Total protein concentrations were determined with a BCA protein assay reagent kit (Pierce/Thermo Scientific, Rockford, IL, USA) according to manufacturer's protocol. Aliquots corresponding to 10 μg total protein were separated by SDS-polyacrylamide gel electrophoresis (5–20% gradient gel) and blotted onto polyvinylidene difluoride (PVDF) membranes. The membranes were blocked for 1 h in Blocking One (Nacalai Tesque) and incubated overnight at 4°C in TBS-T containing 5% bovine serum albumin and monoclonal rabbit anti-Stat3 antibody (1:1000) (Cell Signaling Technology, Danvers, MA, USA) or for 1 h at room temperature in Blocking One containing monoclonal mouse anti-β-actin (1:1000) (Sigma-Aldrich). Subsequently, the membranes were incubated for 1 h with HRP-conjugated antibodies (1:3000) (Amersham Biosciences, Buckinghamshire, UK). Stat3 and β-actin bands were visualized using the ECL Prime kit (Amersham Biosciences) and ImageQuant LAS 4000 (Fuji Film).

### Luciferase reporter assay

Huh-7 cells were seeded at 8 × 10^3^ cells/well in 96-well white plates (Corning) containing 10% FBS/DMEM. After 24 h incubation in a 5% CO_2_ incubator, firefly luciferase expression plasmid pGL4.50 (Promega) was added with or without 9 mM CaCl_2_ in the medium. CalPhos Mammalian Transfection Kit (Z1312N; Takara Bio Inc.) was used as a positive control for plasmid transfection according to the manufacturer's protocol. After 48 h, firefly luciferase activity was measured using the ONE-Glo Luciferase Assay system (Promega) and microplate reader Gemini EM (Molecular Devices).

### Dynamic light scattering (DLS) study

CaCl_2_ or MgCl_2_ solution was added with or without ApoB-10177-BNA (13) into 10% FBS/DMEM or DMEM with antibiotics/antimycotics (Sigma-Aldrich). The average particle size and polydispersity index (PDI) of the medium were measured using a Zetasizer (Nano ZS; Malvern Instruments, Southborough, MA, USA).

### Transmission electron microscopy (TEM) analysis

For negative staining, the samples were absorbed onto carbon-coated copper grids (400 mesh) and stained with 2% phosphor tungstic acid solution (pH 7.0) for 10 s. Observation and imaging was performed on the samples using transmission electron microscopy (TEM) (JEM-1400Plus; JEOL Ltd, Tokyo, Japan) at an acceleration voltage of 80 kV. Digital images (2048 × 2048 pixels) were taken with a CCD camera (VELETA; Olympus Soft Imaging Solutions GmbH, Münster, Germany). All TEM experiments were conducted by Tokai Electron Microscopy, Inc. (Aichi, Japan).

### Statistical analyses

Statistical comparisons of results were performed by student's *t*-tests.

## RESULTS

### Ca^2+^ potentiates the activity of 2′,4′-BNA-modified antisense oligonucleotides *in vitro*

To facilitate our methodology development, we employed our robust BNA technology optimized for antisense therapeutics (17,18); this approach allowed us to investigate the effect of CEM on the activity of 2′,4′-BNA-based ASOs. We constructed a cell line (which we designated ZsG-N1–2R/HLE) consisting of human hepatoma HLE cells stably expressing both ZsGreen1 and DsRed reporters. This line enabled us to quantitatively monitor gene silencing by fluorescence measurement. The ZsG-N1–2R/HLE cells were treated by incubation for 4 days with a ZsGreen1 ASO (ZsGN1–120-BNA (15); Supplementary Table S1) in the presence of various concentrations of CaCl_2_ or MgCl_2_. Surprisingly, the knockdown activity of ZsGN1–120-BNA (15) was enhanced by CaCl_2_ in a concentration-dependent manner, while MgCl_2_ did not produce a similar enhancement (Figure 1A). Maximal activity was observed at 9 mM CaCl_2._ Dose-response curves revealed the overwhelming knockdown-enhancement effect of incubation with CEM + ASO (Figure 1B). We thus confirmed incubation with oligonucleotides in medium supplemented with 9 mM CaCl_2_ as a standardized CEM method. We further assessed changes in knockdown activity by means of a time course (Figure 1C). Importantly, a significant knockdown was observed in the first 24 h. This earlier response indicates the advantage of our CEM method over the free-uptake method, consistent with previous *in vivo* results reported elsewhere (10,19).

### The CEM method also works for endogenous targets and for multiple types of cells with limited toxicity

Next, to demonstrate the general utility of the CEM effect on 2′,4′-BNA-based ASOs, we developed ASOs targeting endogenous apolipoprotein B (*ApoB*) mRNA, a known drug target for homozygous familial hypercholesterolemia. A human hepatoma cell line, Huh-7, was incubated with an *ApoB*-targeting ASO (ApoB-10177-BNA (13), Supplementary Table S1) (19) at 100 nM for 24 h in the presence of CaCl_2_. *ApoB* mRNA levels were then quantified by means of qRT-PCR. As expected, the knockdown activity of *ApoB* by ApoB-10177-BNA (13) was enhanced by CaCl_2_ in a concentration-dependent manner (Figure 1D), in agreement with the results obtained above for an exogenous target in a different cell line. Knockdown by ApoB-10177-BNA (13) demonstrated clear dose dependency in the presence of 9 mM CaCl_2_ (Figure 1E); no other metal chlorides examined here improved the potency of ASOs (Supplementary Figure S1).

Survivin mRNA has been reported to be expressed in many cancers (20). To further prove the versatility of the CEM method, we applied CEM to three additional cell lines (human cervical cancer HeLa cells, human embryonic kidney HEK293 cells and adenocarcinomic human alveolar basal epithelial A549 cells), to determine if suppression of Survivin mRNA is achieved with a known clinical phase Survivin-targeting ASO (Survivin-BNA (16), Supplementary Table S1) (21). HeLa, HEK293 and A549 cell lines were incubated with a Survivin-BNA (16) at various concentrations for 24 h with 9 mM CaCl_2_. As expected, Survivin mRNA expression was down-modulated in a concentration-dependent manner and all cell lines were responsive to the CEM method (Supplementary Figure S2). The observation that CaCl_2_ up to a dose of 20 mM does not cause severe cytotoxicity regardless of the presence or absence of ASO was confirmed for up to 96 h in various human cell lines (Supplementary Figure S3). Thus, we confirmed that the CEM method is applicable to both endogenous and exogenous targets as well as different cell lines with very limited cytotoxicity.

To assess if CEM was also effective in primary cells, murine hepatocytes isolated from 7-week-old C57BL/6J male mice were plated in 96-well plates. After 24-h incubation, ApoB-10177-BNA (13) was added to the medium with or without 9 mM CaCl_2_ and incubated for an additional 24 h. Cells were harvested and lysed and total RNA was subjected to qRT-PCR or cell viability was analyzed. The assay revealed that CEM also potentiated the activity of ApoB-10177-BNA (13) in murine hepatocytes, however, the basic nature of poor cell viability of primary cultured cells may limit the utility (Supplementary Figure S4).

### The CEM method accurately predicts *in vivo* activity of ASOs

We next investigated whether the CEM technique provides superior *in vitro* screening compared to conventional lipofection and free-uptake. Fourteen different ASOs targeting the ZsGreen1-encoding gene were transfected into ZsG-N1–2R/HLE cells using lipofection, free-uptake or CEM method. Surprisingly, the CEM method was revealed to have a strong correlation with the free-uptake method (*R*^2^ = 0.936) (Figure 2A), while a poor correlation was observed between CEM and lipofection (*R*^2^ = 0.0565) (Figure 2B). Considering the compatibility between free-uptake and *in vivo* activity (4,10,11,22), the CEM method assay results may serve as a surrogate for determining the *in vivo* activities of ASOs. To confirm this hypothesis, we directly assessed the correlation between *in vitro* and *in vivo* activity with CEM. These experiments employed 10 different 13-mer 2′,4′-BNA-based ASOs designed to target endogenous apolipoprotein B-encoding (*ApoB*) mRNA (Supplementary Table S1). We analyzed the knockdown activities of the ApoB-ASOs using the CEM or lipofection methods in Huh-7 cells, as well as *in vivo* activity in mouse liver. As expected, a high correlation between *in vitro* and *in vivo* activity of the ASOs was observed with CEM (*R*^2^ = 0.854) (Figure 2C). On the other hand, there was a lower correlation between *in vitro* and *in vivo* activities with lipofection (*R*^2^ = 0.162) and it was revealed that conventional lipofection is more likely to have a potential for false-positive detection (Figure 2D, shown as black diamonds).

### Ca^2+^ has minimal impact on subcellular localization pattern of ASOs but enhances uptake of ASOs

**Figure 3.**
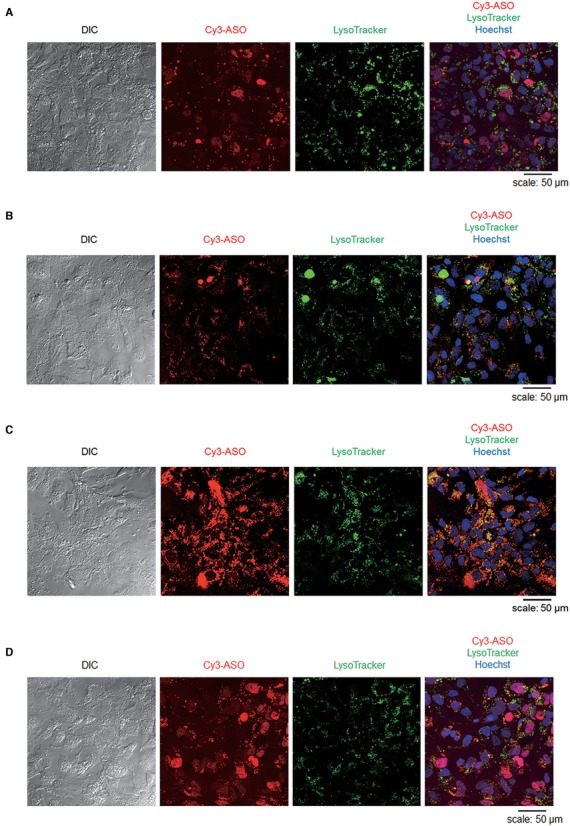
CEM effect on subcellular localization of 2′, 4′-BNA ASO. (**A**) Huh-7 cells were lipofected with Cy3-labeled *ApoB*-ASO at 100 nM using Lipofectamine 2000 (Life Technologies). After 24 h of incubation, nuclei were stained with 0.5 μM Hoechst 33342 (Life Technologies) for 30 min, following by staining of lysosomes with LysoTraker^®^ Green DND-26 (Life Technologies) at 75 nM for 30 min at 37°C. After staining, the medium was replaced with HBSS and the cells were observed using a confocal laser scanning microscope (Leica, TCS SP5). (**B** and **C**) Huh-7 cells were treated with Cy3-labeled *ApoB*-ASO at 500 nM with or without 9 mM CaCl_2_. After 24 h of incubation, the subcellular localization of Cy3-labeled *ApoB*-ASO was visualized by confocal microscopy. (**D**) Huh-7 cells were transfected with the CalPhos™ Mammalian Transfection kit according to manufacturer's procedure. After 24 h of incubation, the subcellular localization of Cy3-labeled *ApoB*-ASO was visualized by confocal microscopy.

We further analyzed the subcellular localization of 2′,4′-BNA-based ASO by determining the difference among three transfection methods using confocal fluorescence microscopy. Huh-7 cells were treated for 1 day with a Cy3-labeled *ApoB*-ASO by means of lipofection, free-uptake or CEM. Lipofection-delivered ASO was observed primarily in the nucleus (Figure 3A). In contrast, the majority of ASO delivered by free-uptake was localized to the perinuclear and lysosomal areas (Figure 3B), as reported previously (7,10). ASO delivered by the CEM method provided localization resembling that obtained by free-uptake, although CEM yielded higher brightness (Figure 3C). We further compared transfection by CEM to that by the conventional calcium phosphate method. Huh-7 cells were treated with Cy3-labeled *ApoB*-ASO using the CalPhos Mammalian Transfection Kit (Takara Bio Inc.). In contrast to the CEM results, ASO delivered by the calcium phosphate method was found primarily in the nucleus, a pattern resembling the subcellular localization observed with lipofection (Figure 3D).

**Figure 4.**
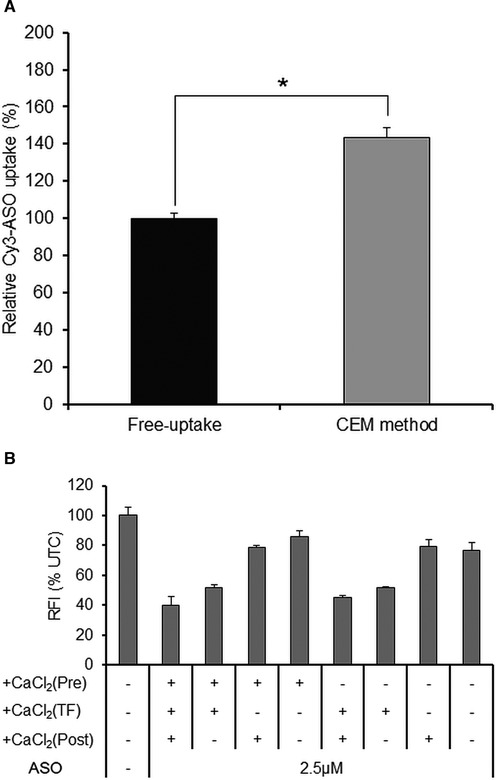
CEM effect on cellular uptake of 2′, 4′-BNA ASO (**A**) Flow cytometric analysis of *ApoB*-ASO delivery into Huh-7 cells. Cells were treated with Cy3-labeled *ApoB*-ASO with or without CaCl_2_ for 2 h in phenol red-free media and washed twice with PBS. Cells (10 000 events) were analyzed using a FACSCalibur flow cytometer and the mean fluorescence intensity of Cy3 was calculated using CellQuest Pro software. Each data point represents the mean ± SD of three independent experiments. Statistical comparisons of results were performed by Student's *t*-tests, *P < 0.0005. (**B**) Addition of CaCl_2_ at different stages of cellular uptake or intracellular trafficking of ZsGreen1-ASO in ZsG-N1–2R/HLE cells. CaCl_2_ was added to the medium before addition of ZsGreen1-ASO (‘Pre’) and/or together with addition of ASO for 7 h (‘TF’), and/or after removal of ASO and washing of the cells (‘Post’). At 72 h after ASO addition, the knockdown activity of the ASO was analyzed. Relative fluorescence intensity was measured and is presented as the percentage relative to the UTC. Each data point represents the mean ± SD of three independent experiments.

To quantitatively analyze the cellular uptake of ASO, we measured the amount of ASO in Huh-7 cells using Cy3-labeled *ApoB*-ASO and flow cytometric analysis. Huh-7 cells were treated with Cy3-labeled *ApoB*-ASO in the presence or absence of 9 mM CaCl_2_ for 2 h in phenol red-free medium; the cells then were washed twice with PBS and fluorescence intensity of Cy3 was quantified using flow cytometry. The mean fluorescence intensity was significantly increased in the presence of 9 mM CaCl_2_ compared to that obtained by free-uptake (Figure 4A). These results confirmed the significantly increased fluorescence observed in CaCl_2_–treated cells compared to that in free-uptake-treated cells.

To further examine if CEM affects the uptake and/or intracellular trafficking of ASO, we next assessed the most efficacious timing for CaCl_2_ exposure, by transfecting ZsGreen1-ASO into ZsG-N1–2R/HLE cells and adding CaCl_2_ at different stages of cellular uptake or intracellular trafficking. Specifically, CaCl_2_ was added to the medium before addition of ZsGN1–120-BNA (15) (‘Pre’ interval) and/or together with addition of ASO for 7 h (‘TF’ interval, expected to be the time of ASO uptake), and/or after removal of ASO and washing of the cells (‘Post’ interval). At 72 h after ASO addition, the knockdown activity of ASO was analyzed. Although the knockdown activity was weak in the absence of CaCl_2_ or when adding CaCl_2_ after removal of ASO, knockdown activity was significantly enhanced when adding CaCl_2_ and ASO concomitantly (Figure 4B). Comparable results were obtained with ApoB-10177-BNA (13) in Huh-7 cells (Supplementary Figure S5). Together, these results suggest that the CEM method works primarily by enhancing the free-uptake step of ASOs, rather than by altering the intracellular trafficking of ASOs.

### CEM method is applicable to siRNAs and phosphorodiamidate morpholino oligonucleotides but not plasmids and cationic lipid/ASO complexes

**Figure 5.**
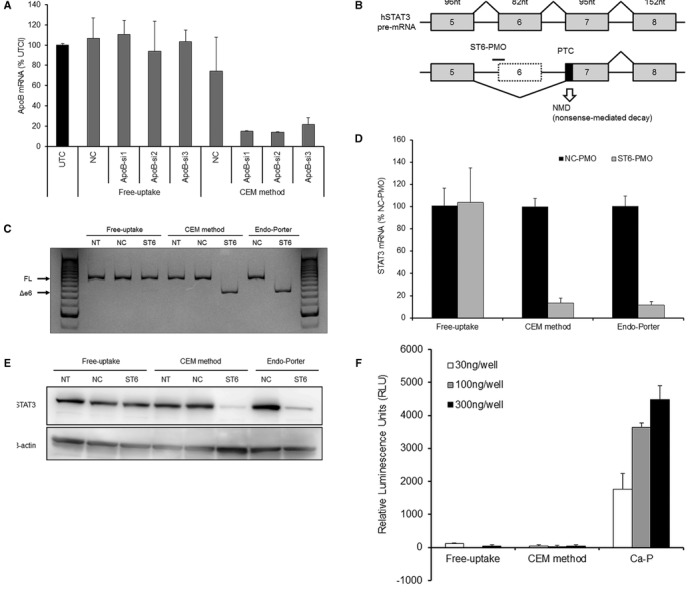
CEM enhances the activity of siRNA and PMO, but not the transfection of plasmid DNA. (**A**) CEM effect on the activity of *ApoB*-siRNAs. Huh-7 cells were treated with 1 μM *ApoB*-siRNAs with or without 9 mM CaCl_2_ for 24 h. *ApoB* mRNA levels were analyzed using qRT-PCR. The relative quantification of *ApoB* mRNA was normalized against expression of the *GAPDH* gene. Each data point represents the mean ± SD of three independent experiments. (**B**) Knockdown of *STAT3* by exon skipping and nonsense-mediated decay. ST6-PMO targeting the boundary region of intron 5 and exon 6 in the human *STAT3* pre-mRNA provides exon 6 skipping and induces RNA degradation by nonsense-mediated mRNA decay. PTC: premature termination codon. (**C**) RT-PCR analysis for *STAT3* pre-mRNA splicing. Huh-7 cells were treated with ST6-PMO with or without 9 mM CaCl_2_. After 48 h, *STAT3* pre-mRNA splicing was visualized by polyacrylamide gel separation of RT-PCR products. FL: full-length, Δe6: exon 6 skipping, NT: non-treatment, NC: negative control. (**D** and **E**) qRT-PCR and western blot analysis of *STAT3* expression. Huh-7 cells were treated with ST6-PMO with or without 9 mM CaCl_2_. After 48 h, *STAT3* mRNA was analyzed using qRT-PCR (D) and STAT3 protein was analyzed by western blot (E). For qRT-PCR, the relative quantification of *STAT3* mRNA was normalized against expression of the *GAPDH* gene. Each data point represents the mean ± SD of three independent experiments. β-actin was the loading control for western blot. Endo-Porter was used as the positive control for PMO transfection. (**F**) CEM effect on the transfection of plasmid DNA. Twenty-four hours after seeding of Huh-7 cells, firefly luciferase expression plasmid, pGL4.50 was added with or without 9 mM CaCl_2_ in the medium. CalPhos Mammalian Transfection kit was used as a positive control for plasmid transfection by the calcium phosphate method (Ca-P). At 48 h after transfection, firefly luciferase activity was measured using ONE-Glo Luciferase Assay system and is presented as relative luminescence units (RLU). Each value is presented as the mean ± SD of three independent experiments.

To assess the broader utility of the CEM method, we tested this technique with other oligonucleotide-based therapeutics. Notably, small interfering RNAs (siRNAs) usually are not synthesized with chemical modifications, but the use of siRNAs for silencing typically requires the use of a carrier-dependent transfection agent (e.g. lipofectamine). On the other hand, PMOs consist of fully modified ASOs having charge-neutral phosphordiamidate linkages, modifications that restrict the use of electrostatic interaction-based delivery vehicles for silencing experiments. To test the applicability of our method for use with siRNAs, we treated Huh-7 cells for 24 h with *ApoB*-targeting siRNAs (Supplementary Table S2) in the presence or absence of 9 mM CaCl_2_. The CEM method improved the potency of all siRNAs despite their fragile nature (Figure 5A). We also confirmed these effects using different genetic targets, cell lines and siRNAs (Supplementary Figure S6). To test the applicability of our method for use with PMOs, we employed the *STAT3* exon skipping model. Specifically, PMOs targeting the boundary region of intron 5 and exon 6 in the human *STAT3* pre-mRNA induce exon 6 skipping, resulting in the induction of nonsense-mediated mRNA decay, reducing the expression of *STAT3* mRNA and protein (Figure 5B) (23). In our experiment, we treated Huh-7 cells with the *STAT3*–targeting PMO (Supplementary Table S3) in the presence or absence of 9 mM CaCl_2_. Notably, *STAT3*-PMO-induced exon skipping was observed after 48 h of PMO exposure only in the presence of CaCl_2_, resulting in a reduction of mRNA and protein levels (Figure 5C, D and E). The activity seen with CaCl_2_ supplementation was similar to that seen using a standard transfection agent (Endo-Porter) designed exclusively for use with PMOs.

We further examined the effect of CaCl_2_ on transfection with naked plasmid DNA. Specifically, 24 h after seeding of Huh-7 cells, the firefly luciferase-expressing vector pGL4.50 was added to the medium with or without the addition of 9 mM CaCl_2_ and luciferase activity was measured after 48 h. Interestingly, CaCl_2_ did not potentiate the introduction of pGL4.50 luciferase expression vector at any of the tested plasmid concentrations, whereas the traditional calcium phosphate method provided a plasmid concentration-dependent increase of luciferase activity (Figure 5F). We also assessed CEM for the transfection efficiency of cationic lipid/ASO complexes. Specifically, ZsG-N1–2R/HLE cells were transfected with each of fourteen ZsGreen1-ASOs using the cationic lipid transfection reagent Lipofectamine RNAiMAX (Invitrogen) with or without the addition of 9 mM CaCl_2_. Notably, CEM had little or no effect on the knockdown activity of each ASO delivered by lipofection (Supplementary Figure S7).

### Enrichment of Ca^2+^ promotes formation of nanoparticles in the medium

Our results suggested that CEM does not directly enhance the activity of various oligonucleotides by particle formation via charge interaction (as expected for Ca^2+^ and the phosphate group of the oligonucleotide), but instead might indirectly accelerate the cellular uptake of naked oligonucleotide. To further elucidate the mechanism of the CEM effect, we investigated whether the inclusion of various concentrations of CaCl_2_ led to the formation of particles when added to the culture medium (DMEM with 10% FBS) in the absence of oligonucleotide. Dynamic light scattering (DLS) demonstrated the stable presence of monodispersed nanoparticles (ϕ ∼ 100 nm) when CaCl_2_ was added at ∼9 mM, while MgCl_2_ formed no such particles at any of the tested concentrations (Supplementary Figure S8a and b). These results indicate the involvement of the nanoparticles in the CEM effect. Particle formation was further investigated under varying medium conditions by using DLS (Table 1). While serum-free DMEM contained no obvious particles, stable monodispersed ∼14 nm nanoparticles were found in FBS-containing DMEM; these particles are thus suggested to be derived from a serum component. We also found that the nanoparticles appropriately-sized for CEM (ϕ ∼ 100 nm) could not be formed either in the absence of 10% FBS or 9 mM CaCl_2._ Interestingly, the presence of ASO does not have a great effect on particle size.

**Table 1.** DLS analysis of culture medium supplemented with CaCl_2_, FBS or ASO

CaCl2	FBS	ASO	Z-Average (d.nm)	Pdl
			mean ± SD	mean ± SD
−	−	−	26.043 ± 4.012	0.830 ± 0.166
−	−	+	43.890 ± 9.211	1.000 ± 0.000
+	−	−	1923.667 ± 163.531	0.056 ± 0.061
+	−	+	1848.333 ± 443.374	0.405 ± 0.230
−	+	−	14.400 ± 0.062	0.353 ± 0.005
−	+	+	14.380 ± 0.070	0.342 ± 003
+	+	−	107.267 ± 1.069	0.135 ± 0.007
+	+	+	106.800 ± 0.625	0.127 ± 0.004

**Figure 6.**
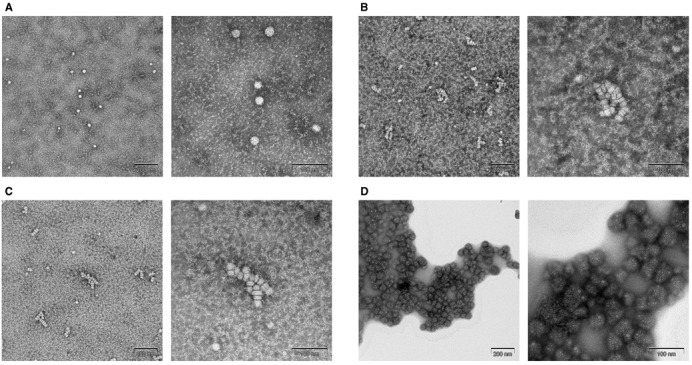
Analysis of culture medium supplemented with CaCl_2_ by negative stain transmission electron microscopy. (**A**) 10% FBS/DMEM + ASO (**B**) the CEM condition (10% FBS/DMEM + 9 mM CaCl_2_) (**C**) the CEM condition + ASO (**D**) DMEM without FBS + 9mM CaCl_2_ + ASO. Bars in left pictures represent 200 nm and the bars in right pictures represent 100 nm.

TEM analysis was successfully employed to visualize these nanoparticles (Figure 6). First, we observed ∼15 nm particles, potential serum components, under the FBS + ASO condition (Figure 6A, Supplementary Figure S9a). Similarly, under the CEM condition (10% FBS + 9 mM CaCl_2_), the 80–100 nm-sized particles were found regardless of the presence or absence of ASO, which is consistent with the DLS analysis. It is particularly worth noting that this 100 nm particle was not one large particle, but rather an ensemble of ∼15 nm-sized nanoparticles (Figure 6B, C, Supplementary Figure S9b and c). Under conditions of CaCl_2_ + ASO in the absence of FBS, the very high surface tension of this solution prevented clear analysis by TEM (Figure 6D, Supplementary Figure S9d). Nonetheless, we observed smaller particles were aggregated into much larger masses in the solution as observed in Table 1.

## DISCUSSION

This report describes a key phenomenon in which Ca^2+^ enrichment of medium potentiates the activity of a broad range of oligonucleotides in a wide range of cell types (Figures 1 and 5, Supplementary Figure S2 and S4). None of the conventional *in vitro* transfection methods permit such universal application. The conventional transfection reagents were originally designed according to the physicochemical characteristics of each oligonucleotide, such as hydrophobicity, charge and size. These reagents are now widely used in the laboratory based on efficient enhancement of the *in vitro* activity of the corresponding oligonucleotides. However, these tailored vehicles often fail to properly predict *in vivo* activity of therapeutic oligonucleotides (Figure 2D), possibly because these reagents facilitate cellular uptake via a pathway distinct from the native route taken by naked oligonucleotides *in vivo*. This lack of correlation impedes the seamless conversion of oligonucleotides from tools for deciphering of biological and pathological mechanisms to clinical drug candidates. In fact, most cultured cells require carrier-assisted transfection for robust transfection by oligonucleotides, while unformulated biologically stabilized oligonucleotides have been shown to be distributed broadly in peripheral tissues, yielding robust systemic effects (24–27). Previous reports implied the involvement of scavenger receptors for liver endothelial uptake of oligonucleotides (28). Primary culture cells accurately reproduce *in vivo*-like free-uptake of naked oligonucleotides *in vitro*, but this uptake is lost 24–36 h after cell isolation (5,6). Koller *et al*. successfully maintained the ability of primary cells to efficiently take up naked chemically modified oligonucleotides by establishing a mouse hepatocellular SV40 large T-antigen carcinoma cell line (MHT) from a specific transgenic mouse (7). These authors reported the involvement of adaptor protein AP2M1 and the irrelevance of clathrin- or cavelin-mediated mechanisms, although these results were not consistent with previous reports (7). Thus, even the molecular mechanisms concerning cellular uptake of naked ASOs, the most widely studied oligonucleotide agents, are still controversial and little discussed currently.

Alternatively, our standardized CEM method was found to better reflect *in vivo* oligonucleotide activity than conventional transfection methodologies (Figure 2). None of the previous *in vitro* screening systems describe such correlation with *in vivo* knockdown activity. We also found that CEM-delivered ASO provided localization resembling that observed with free-uptake, but showed distinctly increased activity (Figure 3). Most mammalian cells can readily accumulate ASOs; however, only a small portion is predicted to be functional without transfection agents. Geary *et al*. reported that there are two different pathways, referred to as bulk non-productive and minor productive uptake pathways, for the uptake of ASO in liver cells (29). In this context, CEM might enhance the availability of accumulated ASOs by saturating non-productive uptake and/or selectively potentiate productive uptake.

We also confirmed formation of highly monodispersed nanoparticles having a size of ∼100 nm when extra Ca^2+^ was included in the medium; such precipitates were not observed in the presence of Mg^2+^, one of the closest metal ions belonging to the alkaline earth group (Supplementary Figure S8a and b). We speculate that these nanoparticles likely play a role in CEM and the formation of these nanoprecipitates may not be directly associated with oligonucleotide charge or physicochemical properties. Thus, it is possible that the nanoprecipitates themselves ‘directly’ accelerate the cellular uptake and/or modify the intracellular behavior of naked oligonucleotides. This narrative is supported by the DLS results shown in Table 1, which indicate that the presence of ASO does not greatly affect particle size; instead, the appropriate-sized nanoparticles for the oligonucleotide transfection (ϕ ∼ 100 nm) can be formed only when both 10% FBS and 9 mM CaCl_2_ coexist in the medium. This indicates the importance of the molecular interaction between FBS and CaCl_2_, rather than with ASO, for the formation of functional nanoparticles. TEM imaging analysis revealed each functional 100 nm-sized particle consists of smaller ∼15 nm nanoparticles, indicating that Ca^2+^ acts as a ‘glue’ to bind serum components together. Similar particulate serum components might function *in vivo*.

Interestingly, CaCl_2_ did not potentiate the uptake of plasmid DNA (Figure 5) despite similarities with the traditional calcium-phosphate method, a technique that depends on the formation of co-precipitates between plasmid DNA and calcium phosphate. Thus, although the CEM method appears applicable to any oligonucleotide independent of the oligonucleotide's net charge and chemical modification pattern, the CEM molecular mechanism described above clearly differs from that of the traditional calcium phosphate plasmid transfection method.

In conclusion, this modality provides a more accurate prediction of the systemic activity of oligonucleotides and is expected to serve as a more cost-efficient and easy-to-access laboratory technique to modulate gene expression. The CEM method is anticipated to provide opportunities to illuminate poorly understood molecular mechanisms of tissue and cellular uptake of naked oligonucleotides.

## Supplementary Material

SUPPLEMENTARY DATA
