# Supplementary material for: Ca2+ enrichment in culture medium potentiates effect of oligonucleotides
Source: Nucleic Acids Res. 2015 Jun 22;43(19):e128. doi: 10.1093/nar/gkv626 (PMC4627064; doi:10.1093/nar/gkv626)
Supplement: SUPPLEMENTARY DATA [file supp_43_19_e128__index.html]

Ca2+ enrichment in culture medium potentiates effect of oligonucleotides — Ca2+ enrichment in culture medium potentiates effect of oligonucleotides — SUPPLEMENTARY DATA 

# Ca2+ enrichment in culture medium potentiates effect of oligonucleotides

## SUPPLEMENTARY DATA

- SUPPLEMENTARY DATA
