# Supplementary material for: Ca2+ enrichment in culture medium potentiates effect of oligonucleotides
Source: Nucleic Acids Res. 2015 Jun 22;43(19):e128. doi: 10.1093/nar/gkv626 (PMC4627064; doi:10.1093/nar/gkv626)

**SUPPLEMENTARY INFORMATION**

**Ca^2+^ Enrichment in Culture Medium Potentiates Effect of Oligonucleotides**

Shin-ichiro Hori^1^,**^†^**, Tsuyoshi Yamamoto^1^,**^†^**,*, Reiko Waki^1^, Shunsuke Wada^1^, Fumito Wada^1^, Mio Noda^1^ and Satoshi Obika^1^,*

^1^ Graduate School of Pharmaceutical Sciences, Osaka University, 1–6, Yamadaoka, Suita, Osaka, 565–0871, Japan

* To whom correspondence should be addressed. Tel: +81 6 6879 8200; Fax: +81 6 6879 8204; Email: obika@phs.osaka-u.ac.jp

Correspondence may also be addressed to Tsuyoshi Yamamoto. Tel: +81 6 6879 8202; Fax: +81 6 6879 8202; Email: t-yam@phs.osaka-u.ac.jp

**^†^** The authors wish it to be known that, in their opinion, the first two authors should be regarded as Joint First Authors.

**SUPPLEMENTAL FIGURE LEGENDS**

**Supplementary Figure 1 | Analysis of the effects of other metals on Huh-7 cells**

Huh-7 cells were seeded in 96-well plates in 10% FBS/DMEM. After 24 h, ApoB-10177-BNA(13) was added in the presence or absence of each metal chloride at 10 mM. However, MnCl_2_, ZnCl_2_, and CoCl_2_ were added at 0.1 mM due to cell toxicity at higher concentrations. After 24 h, the knockdown activity of the ASO was analyzed.

**Supplementary Figure 2 | Analysis of the CEM effects on several other cell lines**

HeLa (a), HEK293 (b) or A549 (c) cells were seeded in 96-well plates containing 10%FBS/DMEM. After 24 h, Survivin-BNA(16) was added, and cells were cultured in medium with or without CaCl_2_. After 24 h, total RNA was extracted and *Survivin* mRNA was quantitated by qRT-PCR. The primers used in this study were specific for the human *Survivin* gene (forward: 5’- AGTCTGGCGTAAGATGATGGATTTG-3’; reverse: 5’- CACAGCAGTGTTTGAAATGACAGG-3’) and for the human *GAPDH* gene. The level of target (*Survivin*) gene expression was normalized to that of *GAPDH*. Each data point represents the mean ± SD of three independent experiments.

**Supplementary Figure 3| Cellular toxicity of calcium chloride**

All of the cells were seeded in 96-well plates containing 10% FBS/DMEM (~50% confluence). After 24 h incubation at 37 °C, CaCl_2_ was added to the medium at concentrations ranging from 0 to 30 mM in the presence or absence of 100 nM ApoB-10177-BNA(13). After 24, 48, 72 and 96 h incubation at 37 °C, the cell viability of Huh-7 (a), HeLa (b), HEK293 (c) or A549 (d) was assessed using WST-8 reagent (Kishida Chemicals, Osaka, Japan) according to manufacturer’s protocol. Each data point represents the mean ± SD of three independent experiments.

**Supplementary Figure 4| Evaluation of the effect and cytotoxicity of CEM method in mouse primary hepatocytes.** Hepatocytes were isolated from 7-week-old C57BL/6J male mice using collagenase perfusion. Isolated hepatocytes were plated on type I collagen-coated 96-well plates at 3 x 10^5^ cells/well. After 24 h, 9 mM CaCl_2_ was added to the medium in the presence or absence of 100 nM ApoB-10177-BNA(13). After 24 h incubation at 37 °C, the knockdown activity of ASO (a) or cell viability (b) was assessed. Each data point represents the mean ± SD of three independent experiments. Statistical comparisons of results were performed by Student’s t-tests, **p*<0.005.

**Supplementary Figure 5| Addition of calcium chloride at different stages of cellular uptake or intracellular trafficking of ApoB-ASO in Huh-7 cells**

Huh-7 cells were seeded in 96-well plates in 10% FBS/DMEM. After 24 h, calcium chloride was added to the medium together with ApoB-10177-BNA(13) for 4 h (“TF”) and/or after removal of ASO and washing of the cells (“Post”). At 24 h after ASO addition, the knockdown activity of the ASO was analyzed. The relative quantification of *ApoB* mRNA was normalized against expression of the *GAPDH* gene. The relative expression of *ApoB* mRNA is presented as the percentage relative to the untreated control (UTC). Each data point represents the mean ± SD of three independent experiments.

**Supplementary Figure 6| Evaluation of knockdown activity of ZsGreen1-siRNA in ZsG-N1-2R/HLE cells**

ZsG-N1-2R/HLE cells were seeded in 96-well black plates in 10% FBS/DMEM. After 24 h, ZsG-siRNA was added at 1 μM in the presence or absence of 9 mM CaCl_2_ in the medium. After 4 days, the fluorescence of ZsGreen1 and DsRed were measured using a SPECTRAmax GEMINI microplate spectrofluorometer. Knockdown efficiency of siRNA was calculated by dividing the fluorescence of ZsGreen1 by that of DsRed. RNAiMAX (Invitrogen) was used as the positive control for siRNA transfection according to manufacturer’s protocol.

**Supplementary Figure 7| CEM effect on the transfection efficiency of cationic lipid/ASO complexes.**

ZsG-N1-2R/HLE cells were transfected with ZsGreen1-ASOs using the cationic lipid transfection reagent Lipofectamine RNAiMAX with or without addition of calcium chloride. After 48 h, the fluorescence of ZsGreen1 and DsRed were measured and knockdown efficiency of each ASO was calculated by dividing the fluorescence of ZsGreen1 by that of DsRed. Each data point represents the mean ± SD of three independent experiments.

**Supplementary Figure 8 | Dynamic light scattering (DLS) analysis of culture medium supplemented with CaCl_2_ or MgCl_2._**

(a and b) 1M stock solution of CaCl_2_ (a) or MgCl_2_ (b) was added to various final concentrations in 10% FBS/DMEM containing antibiotics. The average particle size and polydispersity index (PDI) of media were measured by using Zetasizer Nano ZS.

**Supplementary Figure 9 | Analysis of culture medium supplemented with CaCl_2_ by negative stain transmission electron microscopy.**

(a) 10% FBS/DMEM + ASO (b) the CEM condition (10% FBS/DMEM + 9 mM CaCl_2_) (c) the CEM condition + ASO (d) DMEM without FBS + 9mM CaCl_2_ + ASO. Bars in left pictures represent 200 nm and the bars in right pictures represent 100 nm.

**Supplementary Figure 1**


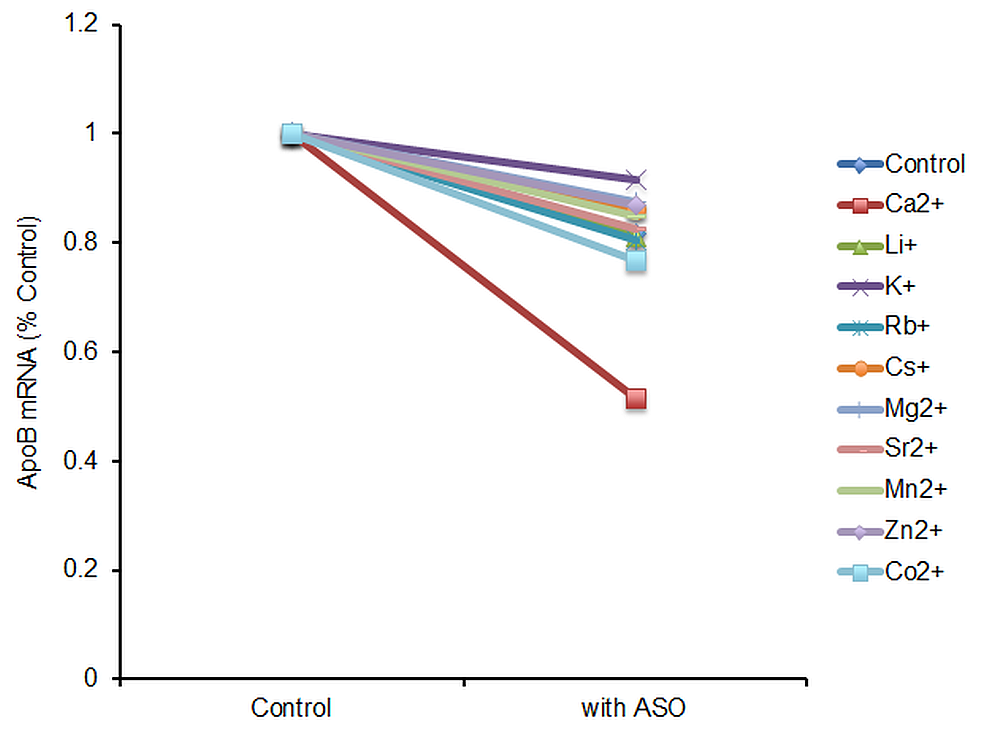


**Supplementary Figure 2**

**(a) HeLa**


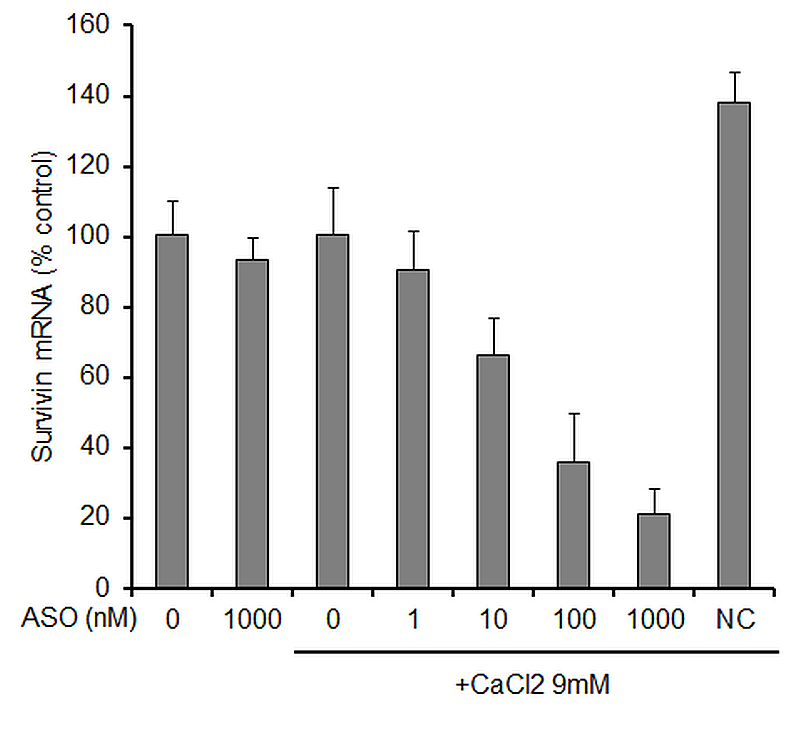


**(b) HEK293**


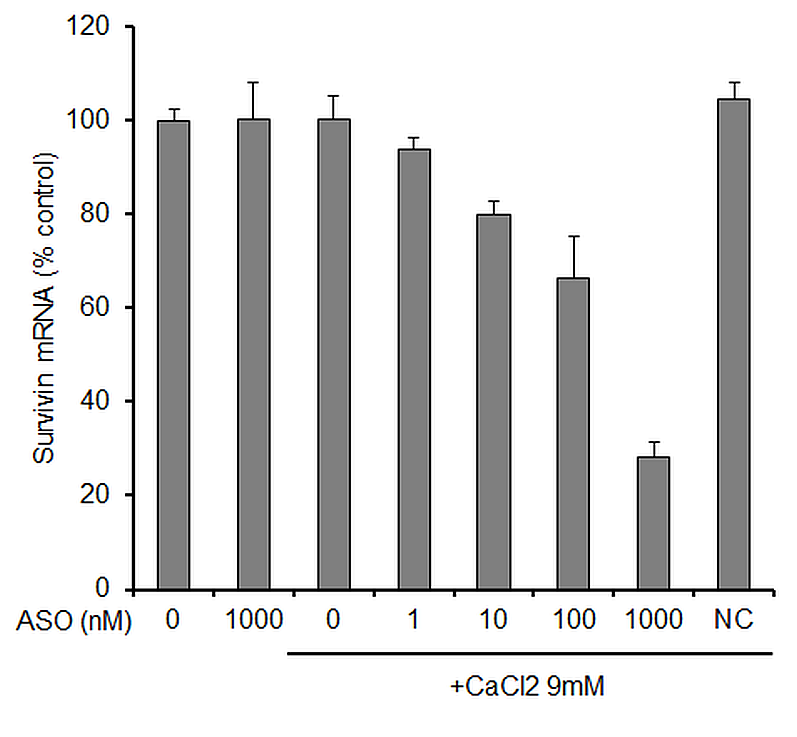


**(c) A549**


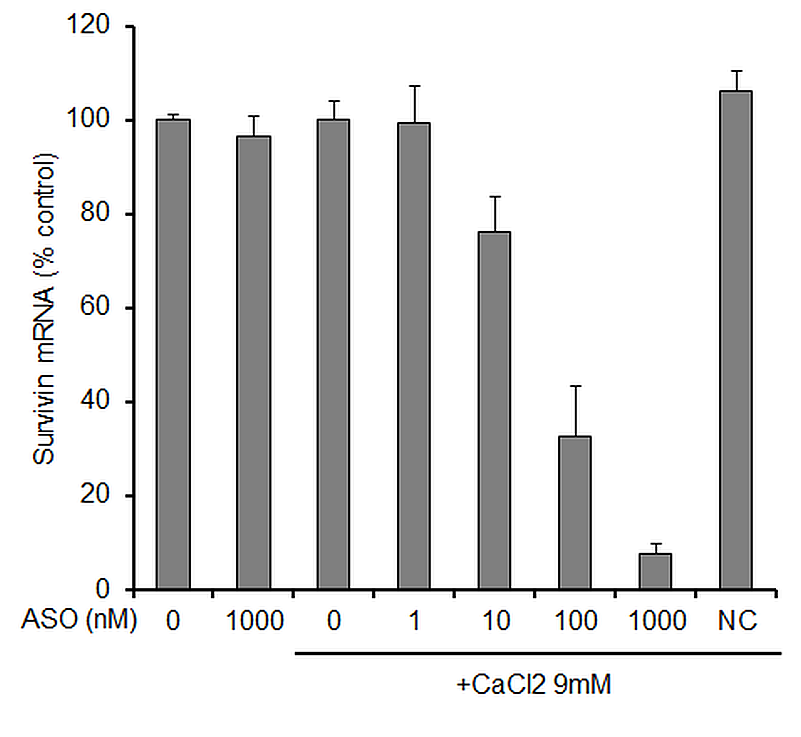


**Supplementary Figure 3**

**(a)**


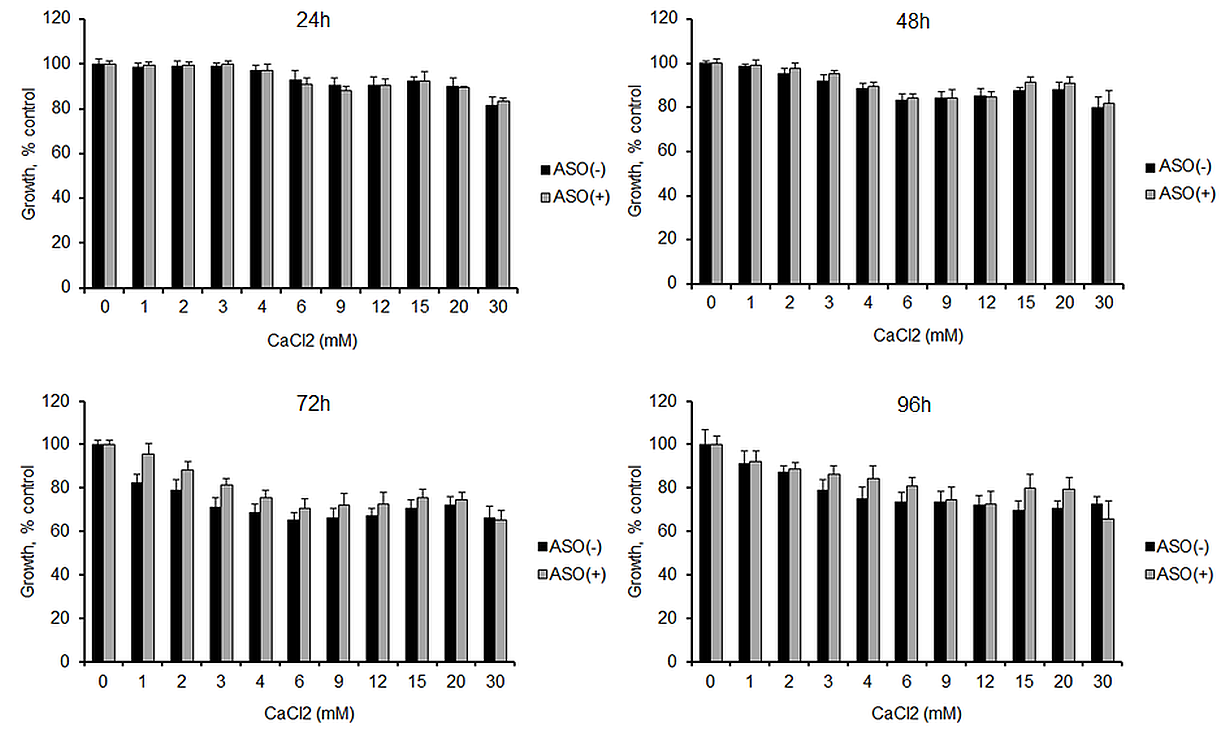


**(b)**


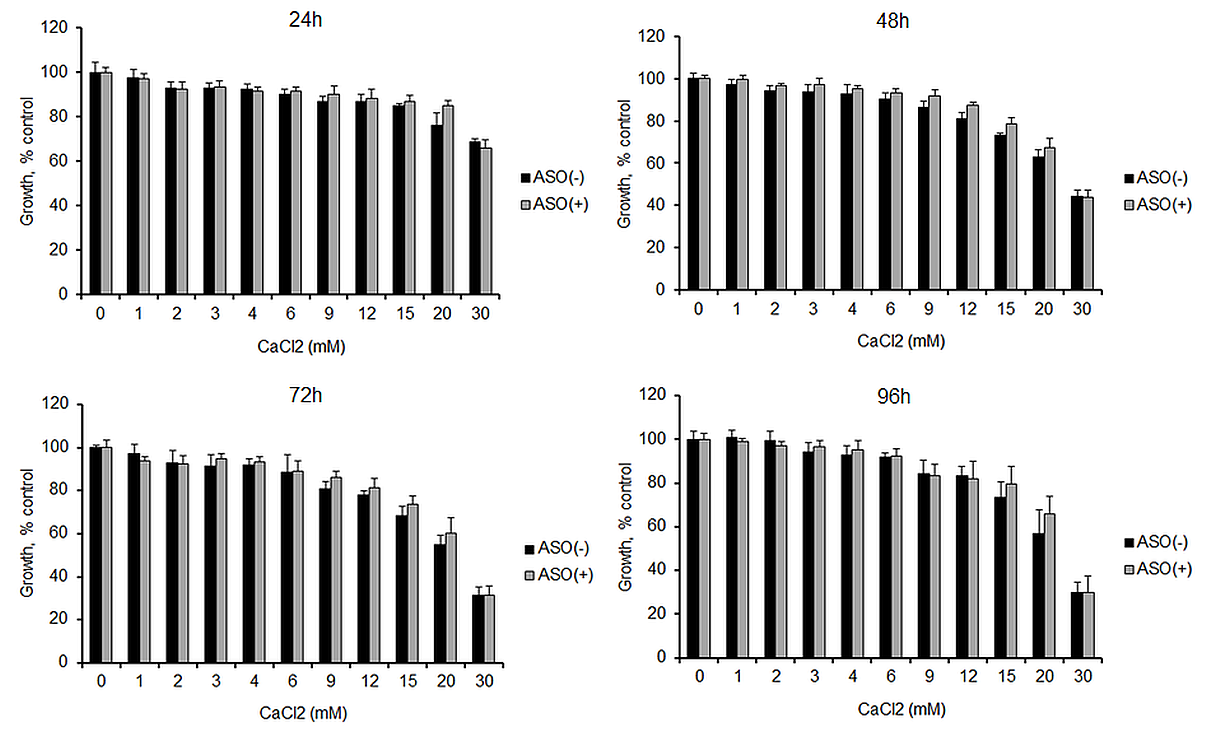


**(c)**


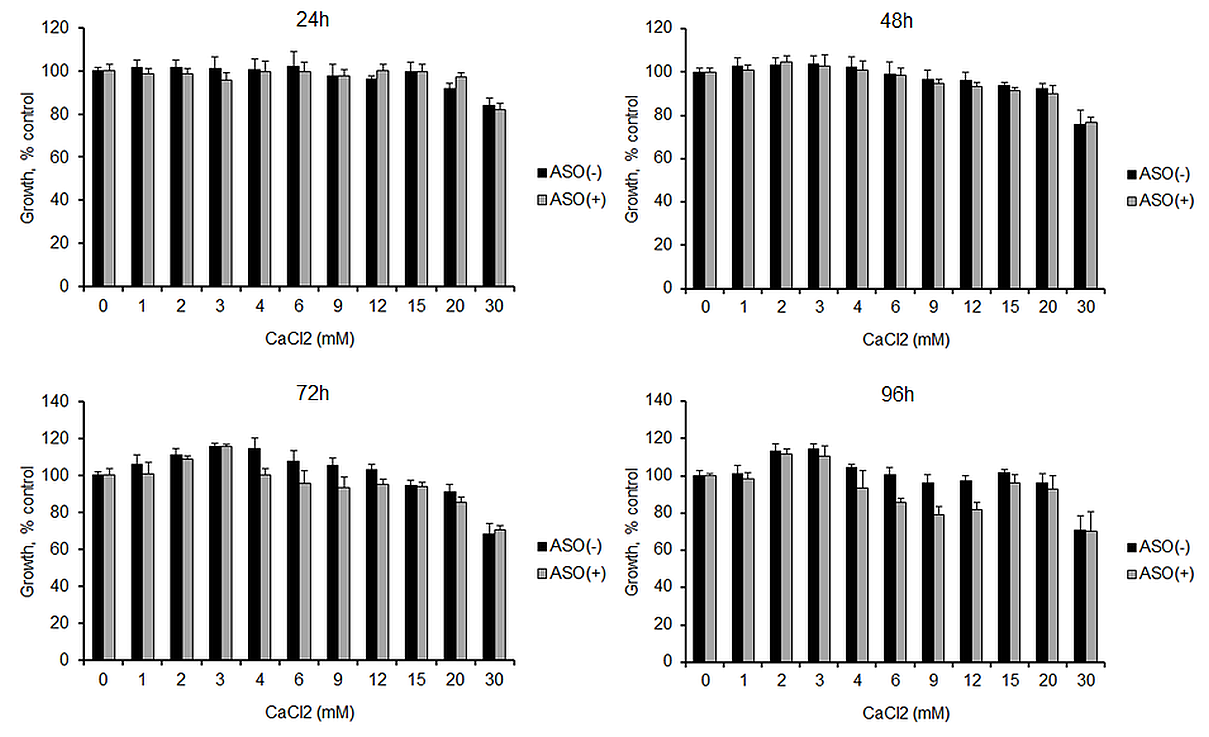


**(d)**


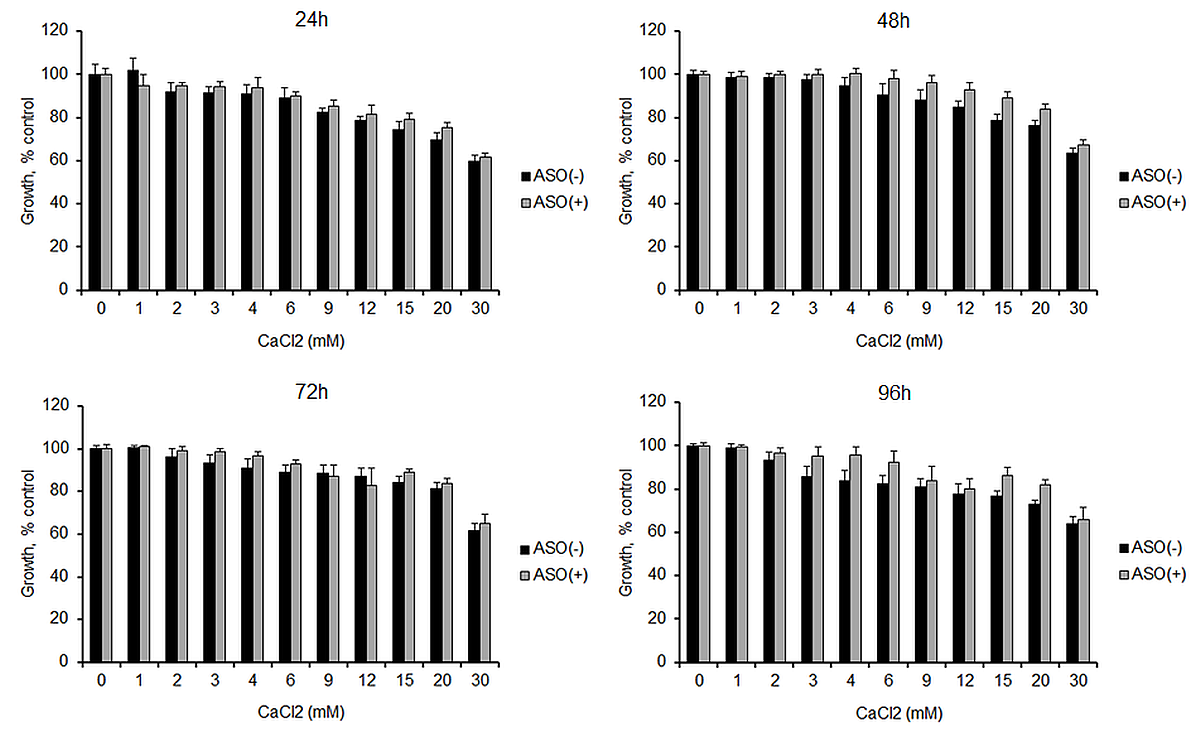


**Supplementary Figure 4**

**(a)**


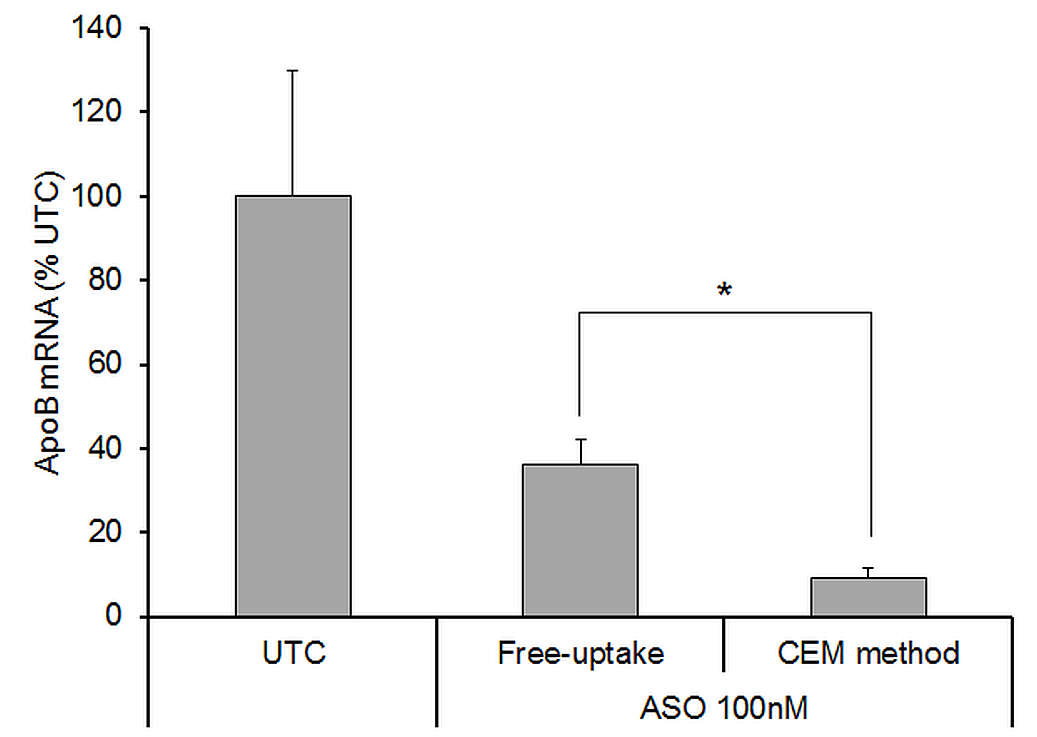


**(b)**


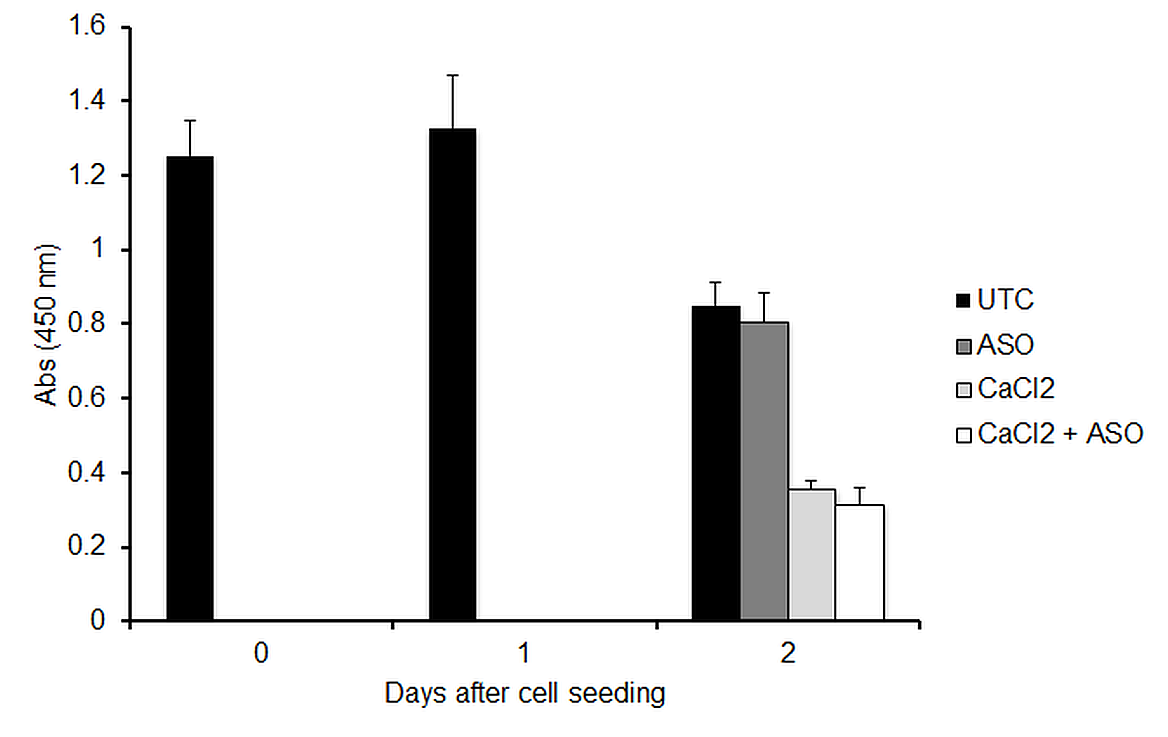


**Supplementary Figure 5**


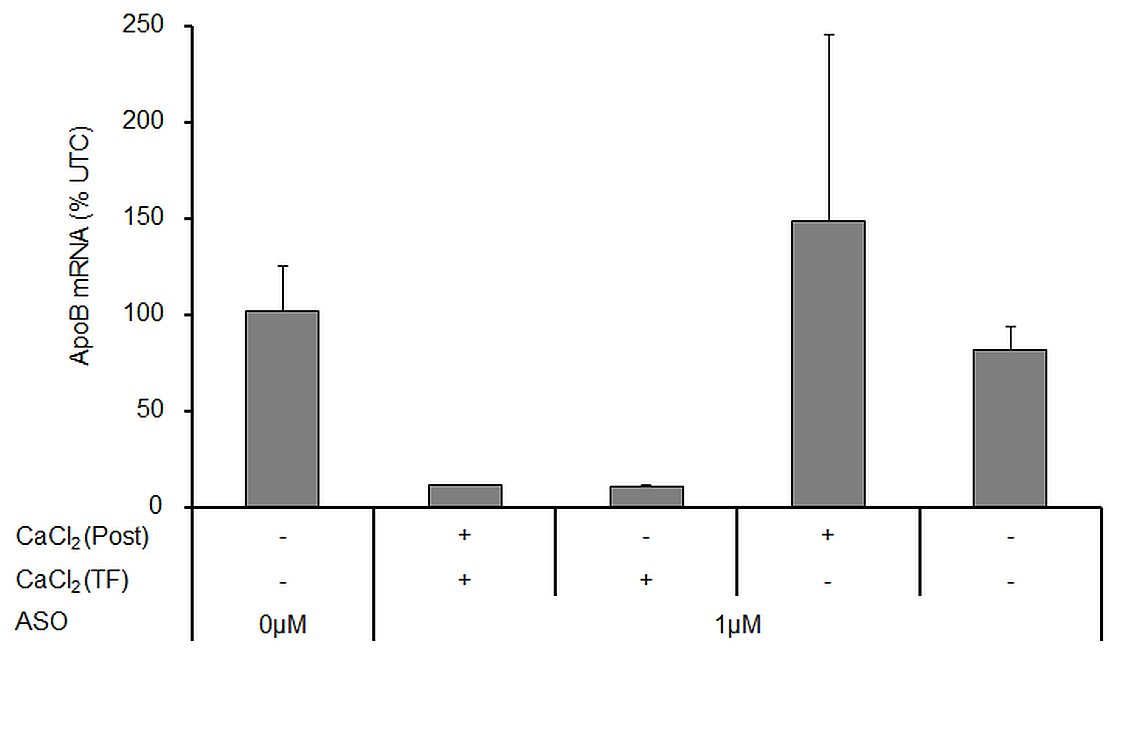


**Supplementary Figure 6**


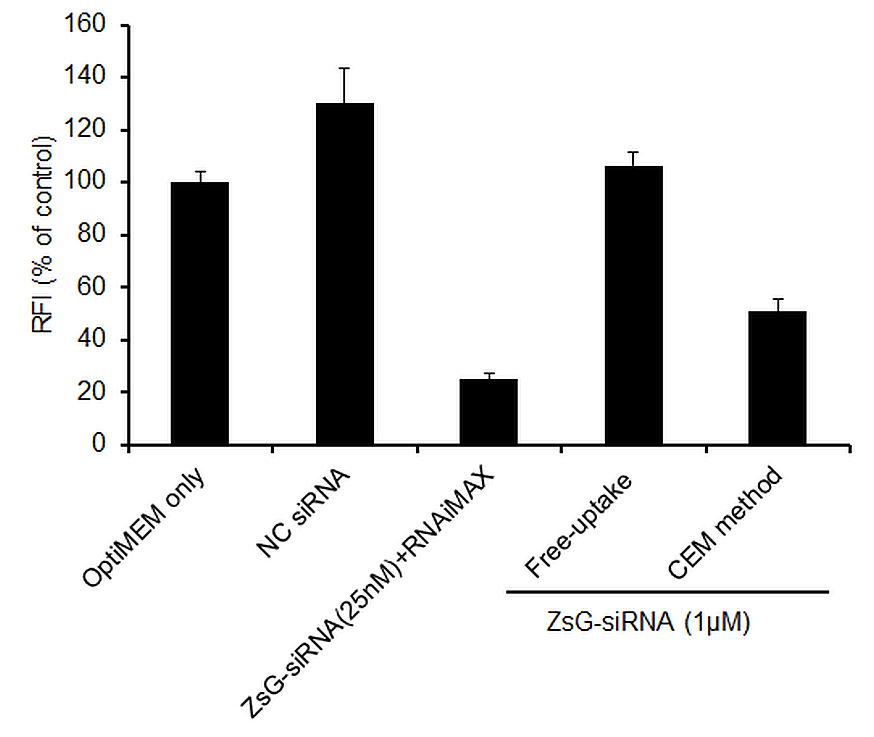


**Supplementary Figure 7**


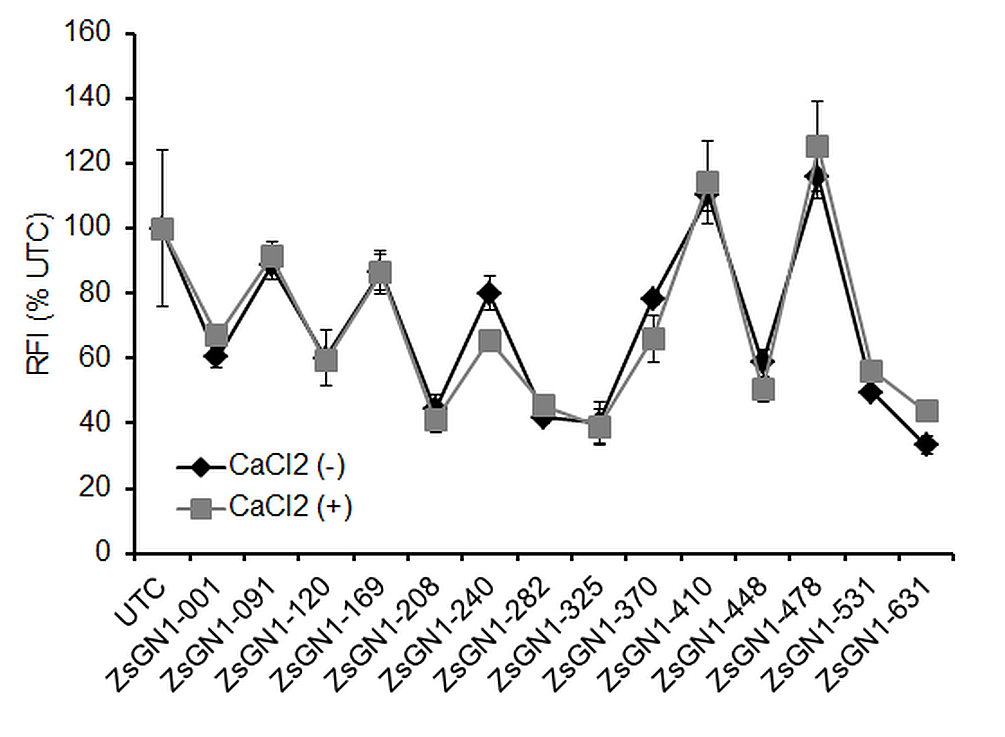


**Supplementary Figure 8**

**(a)**


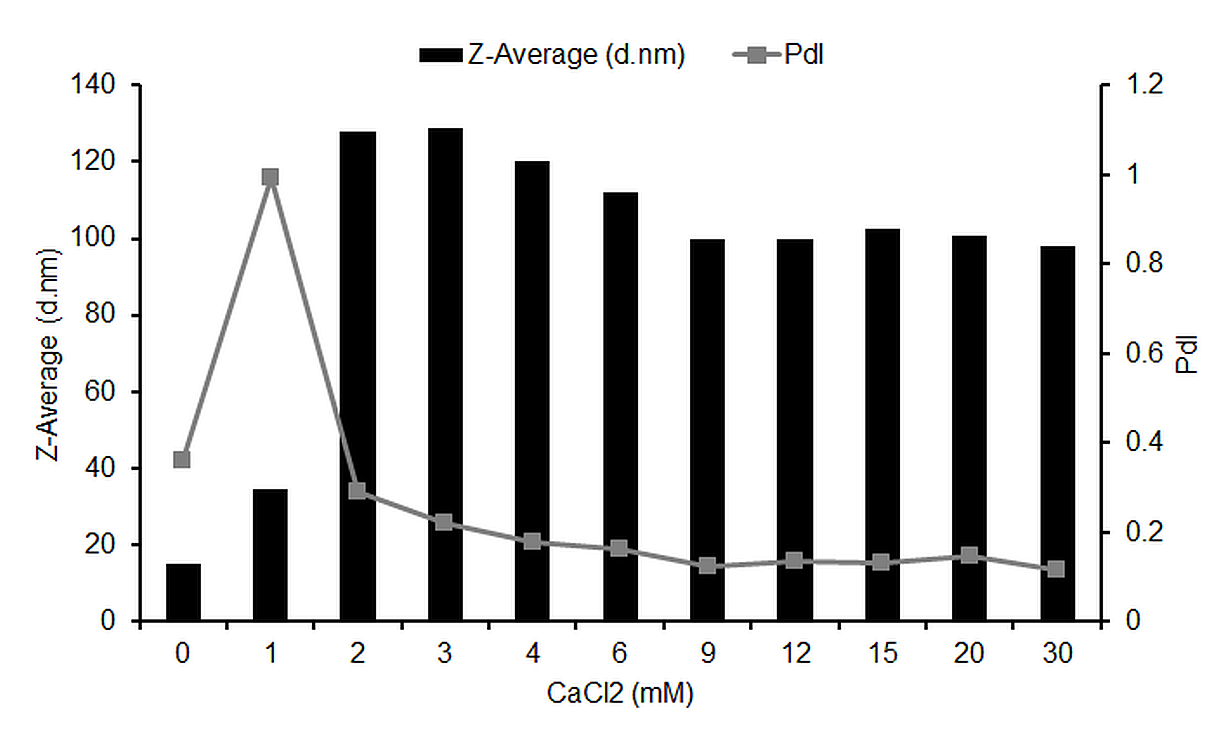


**(b)**


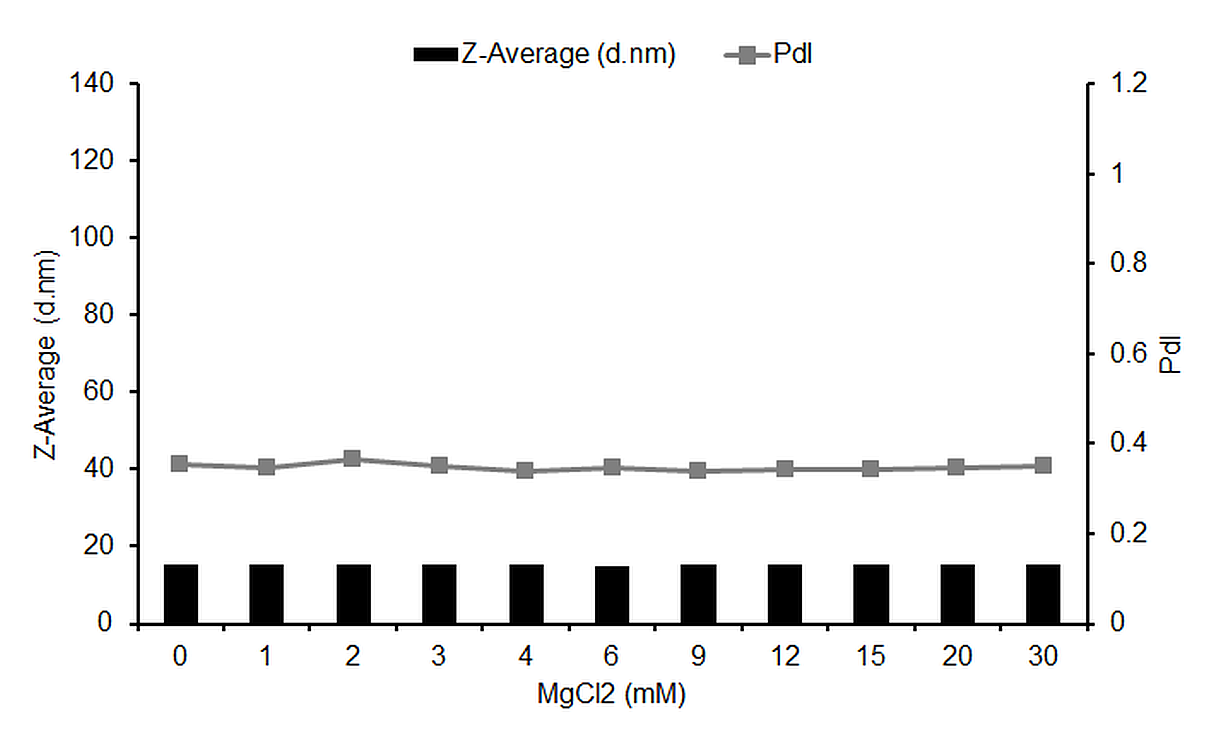


**Supplementary Figure 9**

**(a)**


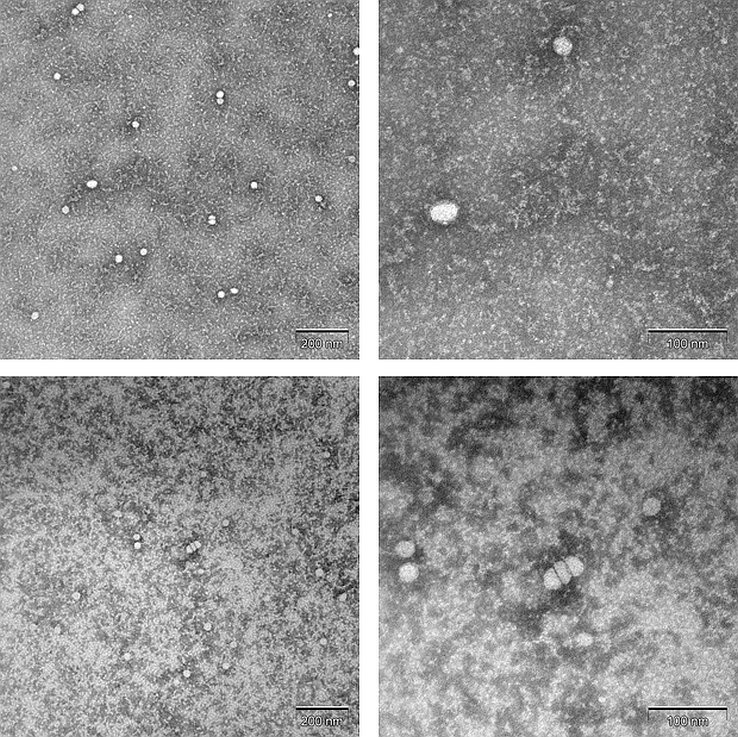


**(b)**


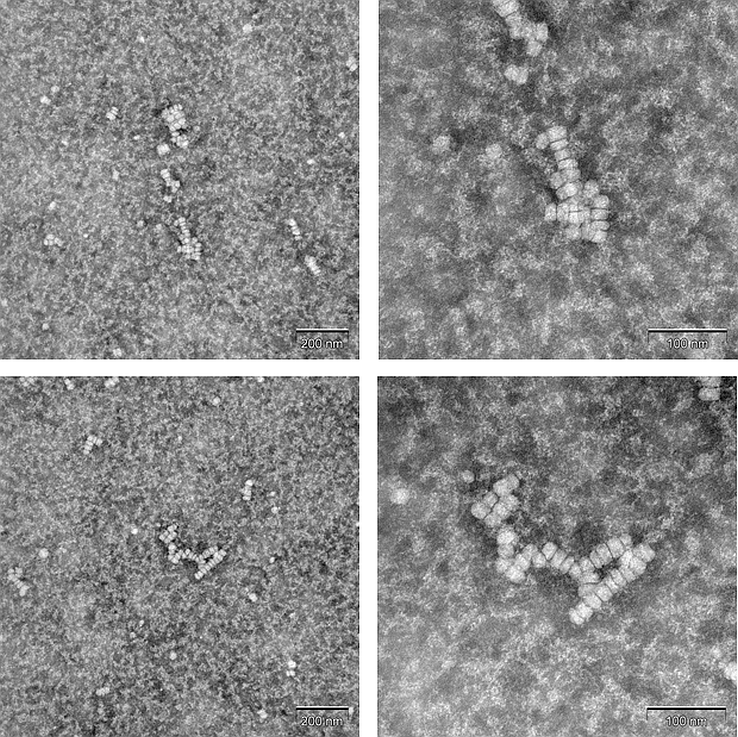


**(c)**


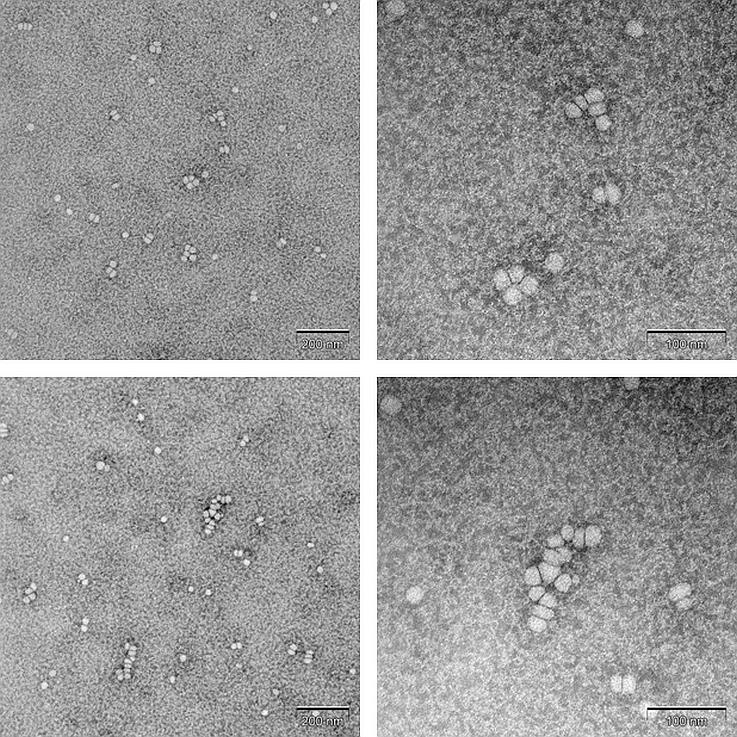


**(d)**


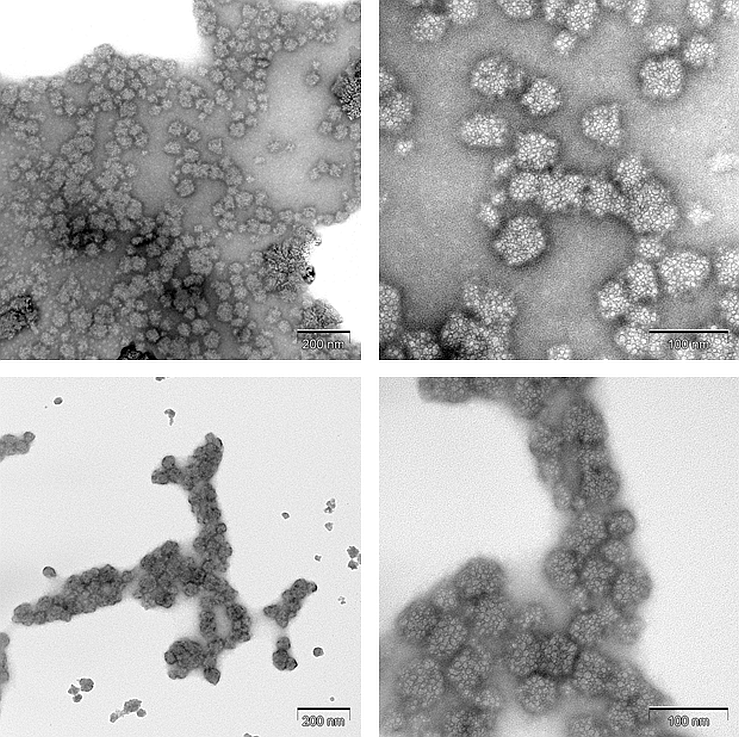


**SUPPLEMENTAL TABLES**

**Supplementary Table 1 | Sequence of 2’ ,4’-BNA/LNA modified ASOs used in this study**

Uppercase: 2’ ,4’-BNA/LNA. Lowercase: DNA. All linkages are phosphorothioated.


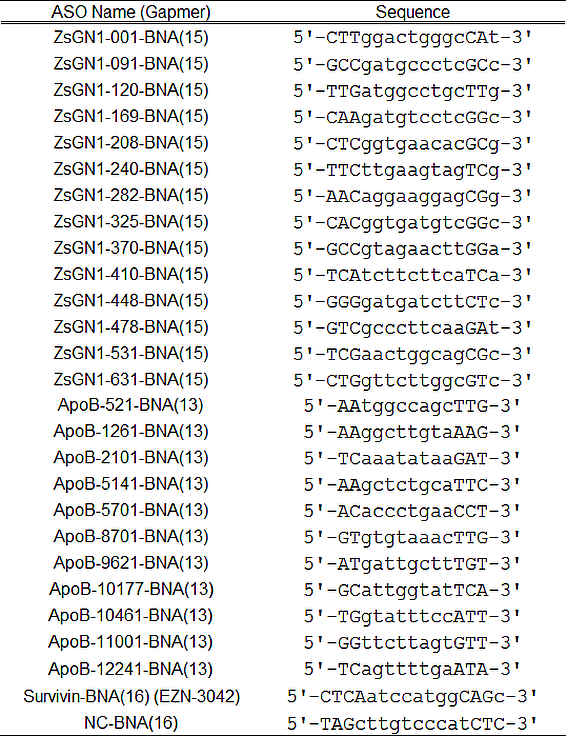


**Supplementary Table 2 | Sequence of siRNAs used in this study**


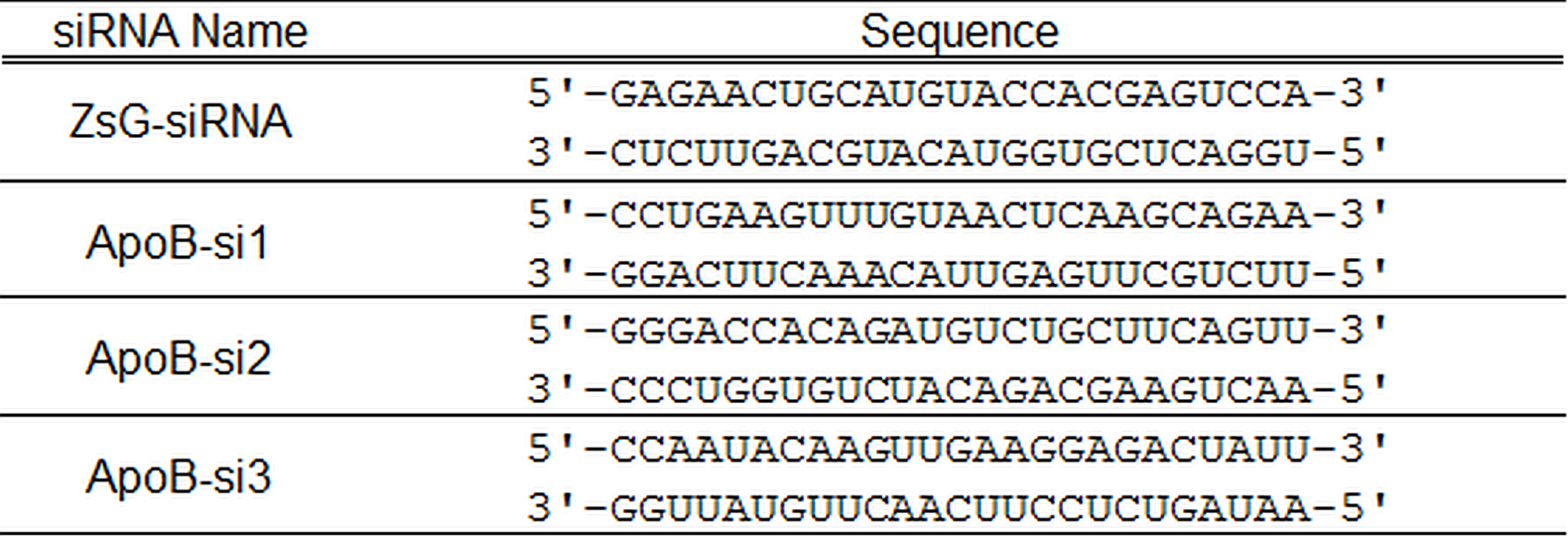


**Supplementary Table 3 | Sequence of PMOs used in this study**


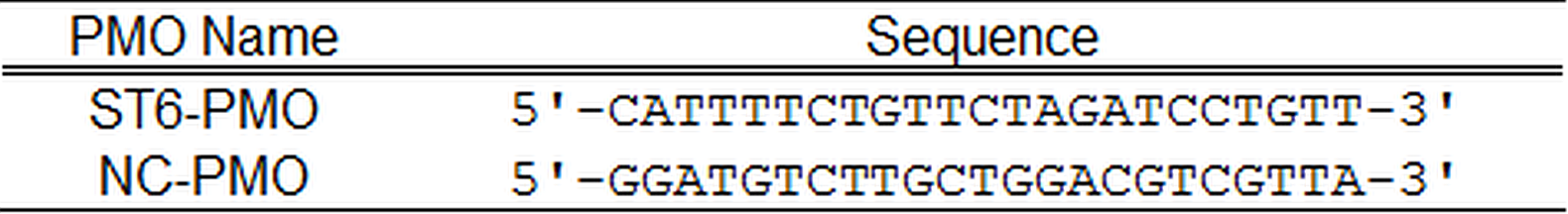

Supplement: SUPPLEMENTARY DATA [file supp_gkv626_nar-01204-met-g-2015-File028.doc]
